# Cation Exchange Protocol to Radiolabel Rare-Earth Nanoparticles with Yttrium-90 for Radiotherapy and for Magnetic Resonance Imaging

**DOI:** 10.1021/acsami.5c05495

**Published:** 2025-06-09

**Authors:** Nisarg Soni, Ana Maria Panaite, Tuhin Samanta, Giulia E. P. Nucci, Emille M. Rodrigues, Teresa Pellegrino

**Affiliations:** 121451 Italian Institute of Technology, via Morego 30, Genoa 16163, Italy

**Keywords:** lanthanide-based nanoparticles, cation exchange, radiolabeling, radiotherapy, MRI

## Abstract

Internal radiation therapy (iRT) is an emerging therapeutic approach based on high-energy radionuclide implants categorized as alpha or beta particles placed directly into the tumor to induce cancer cell damage. This work focuses on the development of a unique approach for incorporating β-emitter yttrium-90 (^90^Y) radionuclides via a cation exchange method into lanthanide-based nanoparticles (NPs), consisting of NaLnF_4_ composition (Ln = Gd, Lu). The proposed method, thanks to the principle of cation exchange, is a straightforward protocol that involves just the mixing of water-stabilized NPs and radionuclides in aqueous environments at room temperature and, upon a short incubation time, enables the exchange of Gd or Lu ions with ^90^Y with high efficiency. The radiotherapeutic effect of cation-exchanged NaLnF_4_:^90^Y is here proven on glioblastoma cell lines with significant cytotoxicity, with the NaLnF_4_:^90^Y NPs, while no intrinsic cytotoxicity was seen for nonradiolabeled NPs at the same material dose. Moreover, in the case of NaGdF_4_ NPs, the gadolinium ions functioning as a T_1_ contrast agents for magnetic resonance imaging (MRI) enables to track the cation exchange protocol by MR signal enhancement during the ion incorporation: indeed, the Y^3+^ replacement with Gd enables the release of Gd^3+^, which enhances the water exposure of Gd ions and, in turn, the enhancement of the T_1_ MRI signal.

## Graphical abstract

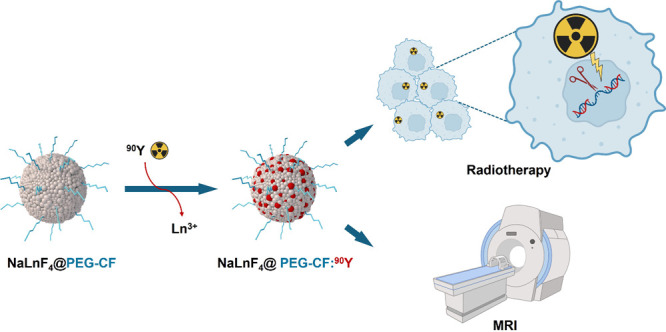

## Introduction

Cancer still represents the primary global cause of death, underscoring the importance of ongoing efforts to uncover minimally invasive treatments while having more effective cancer therapeutic action. Radiation therapy has been proven to be an effective method for treating cancer, with over half of all cancer patients receiving it as either a standalone treatment or in conjunction with other therapies.^1^ The two primary forms of radiation therapy are external beam therapy, in which the tumor is exposed to radiation from an external source, and radionuclide-based iRT, where a radioactive material is placed inside or close to the tumor so that ionizing radiation emitted from radionuclides can cause double-strand breaks in DNA molecules of the tumor cells and, eventually, leading to tumor destruction^2^ while having relatively few side effects.^3^

For iRT, β-Particle emitters, such as iodine-131 (^131^I), yttrium-90 (^90^Y), rhenium-188 (^188^Re), lutetium-177 (^177^Lu), gold-198 (^198^Au), samarium-153 (^153^Sm), holmium-166 (^166^Ho), copper-64, and copper-67 (^64^Cu, ^67^Cu), emit β^–^ particles that can travel only a limited distance (maximum range in soft tissue is about 11 mm), does limit off-target effects.^4,5^ Moreover, the relatively short half-life of these radionuclides ensures a low residence time of the radioactivity in the body. ^90^Y, for instance, is the most widely clinically used radiometal among all the pure β-emitters since it bears a short half-life of 63 h and very high-energy β-particle (*E*_max_ = 2.27 MeV, *E*_mean_ = 939 keV, which makes it an ideal candidate for tumor treatment (see Table S1 for a short summary of the properties).^6^ ^90^Y-loaded microspheres have been applied clinically, but their large size (20–60 μm) limits their use to localized treatment, excluding applications in metastatic cancers.^7,8^ Another alternative solution is represented by antibodies tagged with ^90^Y using chelators like DOTA molecule and also known as tetraxetan, corresponding to the 2,2′,2″,2‴-(1,4,7,10-tetraazacyclododecane-1,4,7,10-tetrayl)­tetraacetic acid molecule.^9,10^ This approach is restricted by the limited amount of ^90^Y that binds the antibodies due to the low number of chelating molecules attached per antibody and the in vivo stability of the ^90^Y-chelate complex itself when exposed to serum proteins.

To overcome these shortcomings, nanoparticle-based platforms were explored to introduce radionuclides either at the NP surface during the NPs synthesis or in a postsynthesis step. Among the available surface strategies, physical adsorption of the radionuclide to the NP surface is one of the simplest, and it was shown to be particularly effective with mesoporous silica-based nanoplatforms, given their exceptional versatility in entrapping various cations, notably including the radionuclides Y-90, Cu-64, Ga-68, In-111, Lu-177, and Zr-89.^11–13^

Another widely applied approach involves the use during the synthesis of NPs of a mixture of radioactive (“hot”) and nonradioactive (“cold”) precursors, resulting in final radiolabeled NPs that incorporate both hot and cold ions.^14^ For instance, Yang et al. employed this method to incorporate ^153^Sm ions into NaLuF_4_:^153^Sm, Yb, Tm upconversion nanoparticles (UCNPs), resulting in a nanoplatform capable of dual imaging modality based on single photon emission computed tomography (SPECT) for nuclear imaging and upconversion luminescence (UCL) imaging.^14^ Moreover, lanthanide-based nanocrystals are compatible with hosting several radionuclides such as ^18^F, ^90^Y, ^124^I, and ^177^Lu, which broadens their scope of theranostics applications. As an example, Liu et al. and Zhou et al. introduced F-18 on the surface of different types of UCNPs with NaYF_4_ and NaGdF_4_ cores,^15,16^ while Lee et al. introduced I-124 on RGD peptide conjugated NaGdF_4_ UCNPs,^17^ and all these kinds of nanoprobes were evaluated for positron emission tomography (PET), MRI, and UCL imaging. Joshi et al. chose physically adsorption of ^177^Lu radioisotope and doxorubicin onto mesoporous silica-coated NaGdF_4_ for combined therapy and SPECT imaging.^18^ Najmr et al. described the synthesis of silica-coated upconverting nanophosphors wherein they mixed Y-90 along with nonradioactive precursors, yielding a multimodal nanoplatform.^13^ Despite their versatility, several radiolabeling approaches face limitations such as low stability, lengthy synthesis procedures, or the need for elevated temperatures and specialized laboratory conditions, which complicate their practical implementation in clinical settings like hospital radiopharmacies.^19,20^ In addition to their radiolabeling potential, lanthanide-based nanoparticles, particularly those doped with paramagnetic ions such as gadolinium (Gd^3+^) ions, offer advantages as MRI contrast agents.^21,22^ While clinical agents like Gd­(DOTA) have been FDA-approved since 1988, concerns over nephrotoxicity in patients with impaired renal function^23,24^ have prompted a shift in research toward Gd^3+^-based nanomaterials. At the same time, new insight into the toxicological profile of Gd ions and other lanthanides is elucidating the main proteins/ions interactions occurring upon blood circulation and their mechanism of toxicity, which has been related to specific organs’ accumulation upon their biodistribution.^25^ Nanoparticles enable to carry higher Gd^3+^ payloads and prolong circulation time, improving signal intensity in target tissues (R_1_, typically given in mM^–1^ s ^–1^).^26,27^ Moreover, the embedding of Gd^3+^ ions into a matrix, such as the crystal structure of inorganic NPs, where they are firmly held, has reduced their leakage from the probe, lowering levels of free Gd^3+^ ions and hence contributing to changing their toxic profile.^28,29^

Cation exchange reactions (CER) are a method to modify NP compositions by performing an ion exchange process on preformed NPs. In a pioneering work by Dong and van Veggel,^30^ this method was first applied to lanthanide fluoride NPs to show the feasibility of changing UCNP composition by incorporating different cations. Other groups have also used CER as a method to tune luminescence properties of lanthanide-doped UCNPs by exchange of double types of cations.^31^

CER has also been selected as the approach for incorporating radionuclides into nanomaterials, which involves postsynthesis incorporation of radioactive cation ions into nanomaterials by replacing nonradioactive cations with radioactive ones while maintaining the anion framework. In a seminal study by Sun et al., Ln ions (Ln = Lu, Yb, Gd, and Tm) in rare-earth-based UCNPs were substituted with the radioisotope ^153^Sm, enabling the resulting radiolabeled NPs to be employed as dual-modality imaging via SPECT imaging and UCL imaging in murine models.^32^ Similarly, CER has been employed on CuS, ZnS, and CdSe/ZnS quantum dots to incorporate positron-emitting isotopes such as ^64^Cu and ^68^Ga ions.^33–36^ Some of these formulations generated self-illuminating nanoparticles capable of PET imaging in conjunction with Cerenkov resonance energy transfer (CRET)-based optical imaging in vivo. While these studies focused primarily on multimodal diagnostic tool development, our group was the first to establish a CER protocol for loading ^64^Cu ions into nanoparticles with an unprecedentedly high specific activity (amount of radioactivity/gram of material) ranging from 2 to 103 TBq·g^–1^.^37^ This advancement not only improved imaging sensitivity but also enabled exploration of the theranostic potential of ^64^Cu-radiolabeled NPs, owing to its dual emission of both β^–^ and β^+^ particles as well as photothermal properties of the host chalcogenide material.

While a few examples in the literature are available for the incorporation of diagnostic radionuclides into nanoparticles, a gap has been observed in the loading of β^–^ emitting therapeutic isotopes, particularly for radionuclides like ^90^Y. The key advantages of using ^90^Y for radiolabeling nanomaterials, over other isotopes are many among which it is worth to highlight that being ^90^Y an FDA-approved pure β-emitter with relative high energy (0.93 MeV) and a medium-short half-life of few days these features make this specific radioisotope ideal choice for radio-tumor treatment and here for the proof of principle of our CER protocol. Therefore, here, we have developed a simple and efficient cation exchange (CE)-based radiolabeling method suitable for the straightforward radioloading of NaLnF_4_ NPs (Ln = either Gd or Lu) with ^90^Y radionuclide. This method allows for the rapid incorporation of ^90^Y under ambient conditions with high yields while also achieving high specific activity labeling, outperforming other radiolabeling techniques. Besides setting the conditions for the quick ^90^Y insertion with the most efficient purification protocol, in the case of NaGdF_4_ NPs compositions, we could also monitor the cation exchange reaction by MRI imaging and T_1_ signal, following the MRI brightness and an increase in the R_1_ signal of the released Gd^3+^ upon exchange. This hallmark work represents the first example and proof of concept of a CE method applied to rare-earth-based NPs as a tool for radioinsertion of pure β^–^ emitters into tiny NPs to be used for iRT to be applied to the radiotherapy treatment of glioblastoma cells.

## Results and Discussion

For developing a radiolabeling protocol, we first synthesized isotropic NaGdF_4_ NPs with an average particle size lower than 10 nm, which is known to be optimal for MRI contrast enhancement (Figure 1A1, Figure S1A).^38^ Moreover, the focus of our study was on cubic-phased NaGdF_4_ since previous studies have shown that they offer enhanced performance in MRI applications. Specifically, they possess higher R_1_ values and an R_2_/R_1_ ratio closer to 1 compared with the hexagonal phased NaGdF_4_, enhancing their effectiveness as MRI T_1_ contrast agents.^39^ Cubic-phased NaGdF_4_ NPs were synthesized by the thermal decomposition method with minor modification to a previously reported work (see Materials and Methods section).^40^ Briefly, the synthesis involved the preparation of the lanthanide trifluoroacetate precursors, followed by their thermal decomposition in a mixture of high-boiling organic solvents and surfactants (e.g., oleic acid, oleylamine, and 1-octadecene), whose ratios were slightly adjusted to enhance particle dispersibility and control size uniformity. Excess NaF, recognizable by the presence of a whitish precipitate, was removed from the NPs solution by washing with an ethanol–water mixture. NaGdF_4_ NPs crystallized in the cubic phase with the *Fm*3̅*m* phase group, well-matched with the standard pattern of bulk NaGdF_4_ crystal structure as shown in XRD patterns (Figure 1B1). The average crystalline size of NaGdF_4_ NPs by XRD was found to be 6 nm as calculated using the Debye–Scherrer equation *D* = (0.9λ/βcosθ), where *D* denotes average crystallite size, λ stands for the wavelength (λ = 1.5418 Å) of incident X-ray, β denotes the corrected full width at half-maximum (fwhm), and θ denotes the diffraction angle (Table 1).^41^ This crystalline size is close to what we observed by transmission electron microscopy (TEM) measurements, which was found to be 6 ± 1 nm. TEM images of oleylamine-capped NaGdF_4_ NPs showed spherical NPs with a monodisperse morphology (Figure S1A).

**Table 1:** Structural Details of NaLnF_4_ NPs^a^

sample	2θ (°)	plane (hkl)	fwhm (°)	fwhm (rad)	θ (rad)	*D* (nm)
NaGdF_4_	28.1	(111)	1.38	0.024	0.245	6
NaLuF_4_	28.4	(111)	0.92	0.016	0.248	9

a XRD crystalline sizes are calculated by the Scherrer equation based on the peaks of the (111) planes of the corresponding samples.

**Figure 1:**
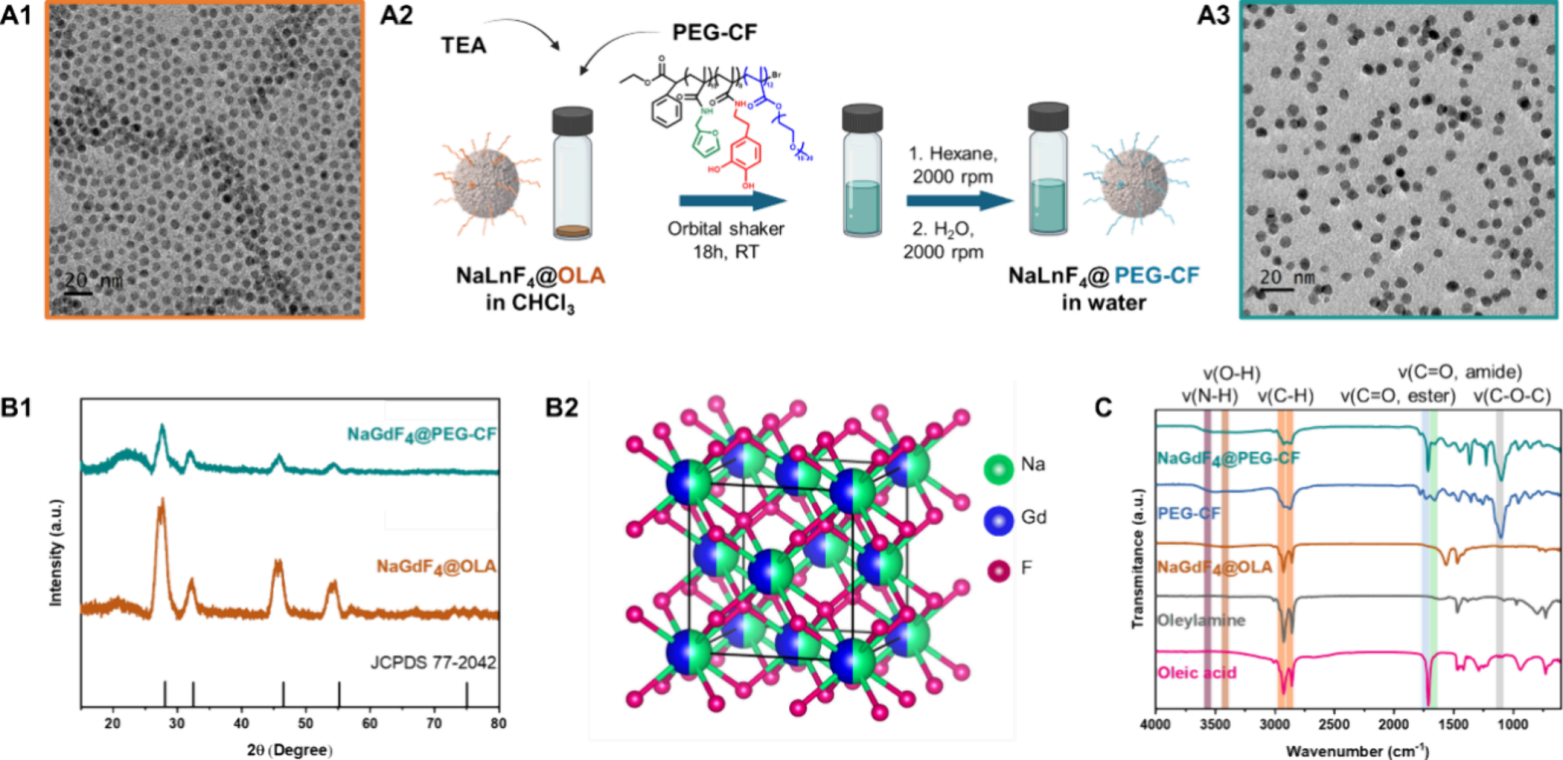
Synthesis and characterization of NaGdF_4_ NPs. (A1) TEM image of the as-synthesized NaGdF_4_ NPs (framed in orange) of size 6 ± 1 nm. (A2) Sketch of the ligand exchange protocol for the water transfer of NaLnF_4_ NPs using the PEG-CF polymer. Created with Biorender. (A3) TEM image of the water-soluble NaGdF_4_@PEF-CF NPs (framed in blue) of similar TEM size 7 ± 1 nm. (B1) XRD pattern of the as-synthesized NaGdF_4_@OLA (B1, orange line) and water-soluble NaGdF_4_@PEF-CF (B1, aquamarine line) compared to the peak patterns of NaGdF_4_ (JCPDS 77–2042). (B2) Unit cell structure of NaGdF_4_ NPs was drawn with visualization for electronic and structural analysis (VESTA) software. (C) FTIR spectra of NaGdF_4_@PEF-CF (C, aquamarine) and PEG-CF ligand (C, blue, where the orange line represents NaGdF_4_@OLA, teal line represents NaGdF_4_@PEF-CF, blue line indicates the PEG-CF polymer, and the gray line indicates oleylamine, and the important peaks are indicated by the shaded regions.

The unit cell of NaGdF_4_ NPs adopts a fluorite (CaF_2_) type structure of high symmetry cation site randomly occupied by Na^+^ and Gd^3+^ ions (see Figure 1B2, which shows the unit cell structure of NaGdF_4_ NPs), consistent with previous reports.^42^ XRD analysis shows that the unit cell structure remains the same before and after the water transfer of NaGdF_4_ NPs with the characteristic peaks observed at 28.1, 32.5, 46.5, 55.1, and 75.0° corresponding to (111), (200), (220), (311), and (331), respectively, matching well with the standard pattern of cubic-phased NaGdF_4_ (JCPDS 70–2042). NaLuF_4_ NPs were also synthesized following a similar protocol, as described in the ESI, and they were found to be 9 ± 1 nm in TEM size (Figure S2A). We observed a similar trend in the XRD pattern for NaLuF_4_ NPs, with the characteristic peaks of NaLuF_4_ NPs observed at 28.4, 32.9, 47.3, 56.1, 58.9, 69.1, and 76.3°, corresponding to (111), (200), (220), (311), (222), (400), and (331), respectively, which is shown in Figure S3. These diffraction peaks match well with the standard pattern expected for cubic-phase NaLuF_4_ (JCPDS 27–0725), confirming the successful synthesis of the desired crystal structure. Moreover, to further support the structural assignments, the 2θ positions, *d*-spacings, corresponding phases, and Miller indices (hkl) of the observed peaks are summarized in Table S2 of the ESI. These results further confirm the phase purity and structural integrity of the NaGdF_4_ and NaLuF_4_ NPs throughout the synthesis. The as-synthesized NaGdF_4_ NPs, coated with hydrophobic oleylamine, were transferred into water following a ligand exchange procedure using a presynthesized multianchoring catechol-based polymer ligand (PEG-CF) composed of several 1,2-dihydroxybenzene­(Catechol) groups for surface anchoring to the NPs surface, equipped with polyethylene glycol units for water-solubility and furfuril amine (F) for possible drug linkage by means of the Diels–Alder reaction (see polymer structure and scheme of the ligand exchange in Figure 1A2). The synthesis and characterization were previously reported by us,^43^ and the scheme of the synthesis of PEG-CF and the ligand exchange protocol details are shown in the Experimental Section). For the exchange, the NaGdF_4_ NPs were simply mixed with polymer ligands (60 ligands/nm^2^) in the presence of TEA (1 mol equivalent to catechol). The resulting solution was left shaking for 18 h on an orbital shaker at RT. The surface-modified NPs were subsequently precipitated by adding an excess of hexane, and the supernatant was discarded after centrifugation, while the precipitate was washed one more time with dissolution in THF and precipitation in hexane. After this step, the NPs collected in the precipitate were easily dissolved in water and concentrated, while the pristine NPs could not be dissolved in water at this step. The same protocol was followed for the water transfer of the NaLuF_4_ NPs. After the water transfer, the NPs retained their morphology and exhibited high monodispersity, with an average TEM size of 7 ± 1 nm. TEM in Figure 1A3, revealed a single layer of NPs spaced far apart from each other. Dynamic light scattering (DLS) measurements indicated a monomodal peak with average hydrodynamic sizes (weighted by intensity) of 26 ± 2 nm for the water-dispersed NPs and a low polydispersity (PdI) index of approximately 0.272 (Figure S4). Therefore, TEM images and DLS analysis confirmed the absence of aggregation in the water-transferred NPs. The multidentate PEG-CF provided a stable polymer shell to the NPs with a recovering yield of NPs measured by elemental analysis of Gd yield recovering up to 99.5% after water transfer. A similar trend was observed in the morphology of NaLuF_4_ NPs, with a recovery yield of NPs here measured by the elemental analysis of Lu of 100% (Figure S2B). The surface features of NaGdF_4_ NPs were analyzed using the Fourier transform infrared spectroscopy (FTIR) technique before and after water transfer (Figure 1C). The FTIR spectra of the as-synthesized NaGdF_4_ NPs were compared to those of the pure oleic acid and pure oleylamine, showing a clear fingerprint of mostly oleylamine with the characteristic bands of terminal methyl asymmetric in-plane stretching νas­(CH_3_) and methyl asymmetric C–H stretching νas­(CH_2_) appeared at 2934 and 2840 cm^–1^, respectively, consistent with reported values for oleylamine-capped NPs.^44,45^ The band around 1580 cm^–1^ was ascribed to the combined motion of NH_2_ scissoring and N–H bending, further supporting the presence of oleylamine. The good match between these signals of FTIR plots for oleylamine and pristine NaGdF_4_ NPs suggests that the oleylamine is present on the NaGdF_4_ NP surface, anchored by the amine group. In the case of a pure multidentate PEG-CF ligand, FTIR spectra show the appearance of characteristic bands of stretching vibrations of O–H and N–H at 3300 and 3500 cm^–1^, respectively. Terminal methyl asymmetric in-plane stretching νas­(CH_3_) and methyl asymmetric C–H stretching νas­(CH_2_) appeared at 2943 and 2885 cm^–1^, respectively. The bands around 1700 and 1600 cm^–1^ suggest the νs­(C=O, ester) and νs­(C=O, amide) respectively, which is consistent with the literature.^46^ The overlap of the FTIR plots of PEG-CF and NaGdF_4_@PEG-CF shows no significant shift of any band, suggesting that NaGdF_4_ NPs are successfully functionalized with the PEG-CF polymer after water transfer. To facilitate the assignment, all major vibrational modes observed for pure ligands, pristine NaGdF_4_ NPs, and NaGdF_4_@PEG-CF are summarized in Table S3 in the ESI.

CER strategy has been used to radiolabel with Cu-64 and Ga-68 ^33,47^ ZnS quantum dots (QDs), and other semiconductor NPs such as CuFeS_2_ NPs.^37^ Here, we set the CER conditions to radiolabel the rare-earth NPs. After the water transfer of NaGdF_4_@PEG-CF NPs via ligand exchange, the first set of CERs was performed with a non-radioactive yttrium salt (YCl_3_), which is named as “cold reaction”. 0.05 M YCl_3_ solution in acetate buffer at pH = 4 was prepared to mimic the same Y^3+^ solution as received in clinic for ^90^YCl_3_ solution, named “hot” solution. In a successful CER condition, 0.25 μmol of YCl_3_ was transferred to 1.5 mL of Eppendorf and diluted to 0.05 M concentration of Y with acetate buffer, followed by the addition of 0.25 μmol of NaGdF_4_@PEG-CF NPs. The CER was investigated at different temperatures (4, 25, 37, and 60 °C), times (5, 10, 20, 30, 60, and 120 min), and molar ratios of Gd^3+^:Lu^3+^ to Y^3+^ (0.1, 1, 10, 20, 30, 40, and 50). Soon after, the reaction mixture was washed and concentrated using centrifuge filters, collecting the NPs on top of the filter, while the filtered solution contained the ions that were freed in solution (Figure 2A). The quantification of Gd and Y or Lu and Y by elemental analyses on the pristine NaGdF_4_ or NaLuF_4_ NPs and upon CER on NP fraction collected on the centrifuge filter (indicated as NaGdF_4_:Y and NaLuF_4_:Y) and in the washing fraction (wash1, wash2, and wash3) were performed by inductively coupled plasma optical emission spectrometry (ICP-OES), thus enabling to calculate the percentage of CE under different experimental conditions (Figure 2B). It is worth knowing that all the CERs were performed at pH 4, as nanoplatforms were unstable at more acidic pH, like pH 1 (Figure S5A,B). At pH values higher than 5, Ln^3+^ ions tend to form hydroxide and precipitate. Also, to ensure the full separation of free cations from the ones bound in the NPs after the CERs, the cation-exchanged NPs, either NaGdF_4_:Y or NaLuF_4_:Y, underwent at least three cycles of amicon filtration. After the CE reaction at 5 min, only a fraction of Y^3+^ ions (below ∼4% of the nominal Y) were found on the NP fraction, while the rest was present in the washing solutions. To optimize the yield of Y associated with the NP fraction, the reaction time of the CER reaction was explored: a series of reactions at different times (5, 10, 20, 30, 60, and 120 min) were performed while keeping fixed the molar ratio Gd^3+^: Y^3+^ = 1:1 (CER 1) at the reaction pH set at 4 (Figure 2C, Figure S5C): with the increase of reaction time, CE percentage and Y^3+^ ions in the NP fraction were increased from 4 to 14%. Indeed, in the case of 5 min reaction, the percentage of cation exchange was 2%, which corresponded to around 4% of Y^3+^ ions trapped in the NaGdF_4_ NPs, and the percentage of Y^3+^ increased to 12 and 14% for CER performed at 60 and 120 min, respectively. Since there was not much significant change in CE percentage between 60 and 120 min, for the “hot” CE reaction with ^90^Y isotope, the reaction time was then fixed at 1 h to guarantee the higher CE and still a reasonable minimal time of exposure for the operator. Having fixed the reaction time to 1 h, the next CER parameter that was optimized was the reaction temperature. The CE reaction was carried out at different temperatures of 4 °C, 25 °C, 37 °C, and 60 °C, and always at a fixed molar ratio of Gd^3+^:Y^3+^ of 1:1, pH 4, and a time duration of 1 h. The CE reaction performed at 4 °C for 60 min showed a low CE percentage and Y^3+^ labeling with ∼7 and ∼8%, respectively. The CE and Y^3+^ labeling percentages increased with the increase in the reaction temperatures. The CE percentages performed at 37 and 60 °C were of ca. 11 and 16%, respectively, while the Y^3+^ percentages associated with the NPs were of ∼14 and ∼15% for the reaction performed at 37 and 60 °C, respectively (Figure 2D). Though CE and Y^3+^ labeling were higher at 60 °C, the CE reaction with the hot Y radioisotope was chosen to be performed at 37 °C to avoid compromising the stabilization of the polymer shell around the particles and the denaturation of biomolecules eventually attached to the NP surface. Next, having selected reaction time and reaction temperature, CER was performed at a molar ratio of Gd^3+^:Y^3+^ different from 1:1 (Figure 2E). It was found that with an increase in NaGdF_4_ NPs (in Gd) compared to Y^3+^ ions, Y^3+^ exchange increased. However, the CE percentage per NP decreased. For instance, in the case of CE reaction with Gd^3+^:Y^3+^ = 50:1 (sample named CER 50), ∼80% of Y^3+^ ions were found on the NaGdF_4_ NP fraction, which only corresponded to 2% of cation exchange per NP (based on the concentration of Gd^3+^ ions). For the CE reaction Gd^3+^:Y^3+^ = 1:10 (sample named CER 0.1), the exchange percentage was very high (∼40%) with a low Y^3+^ labeling percentage (∼2%).

**Figure 2:**
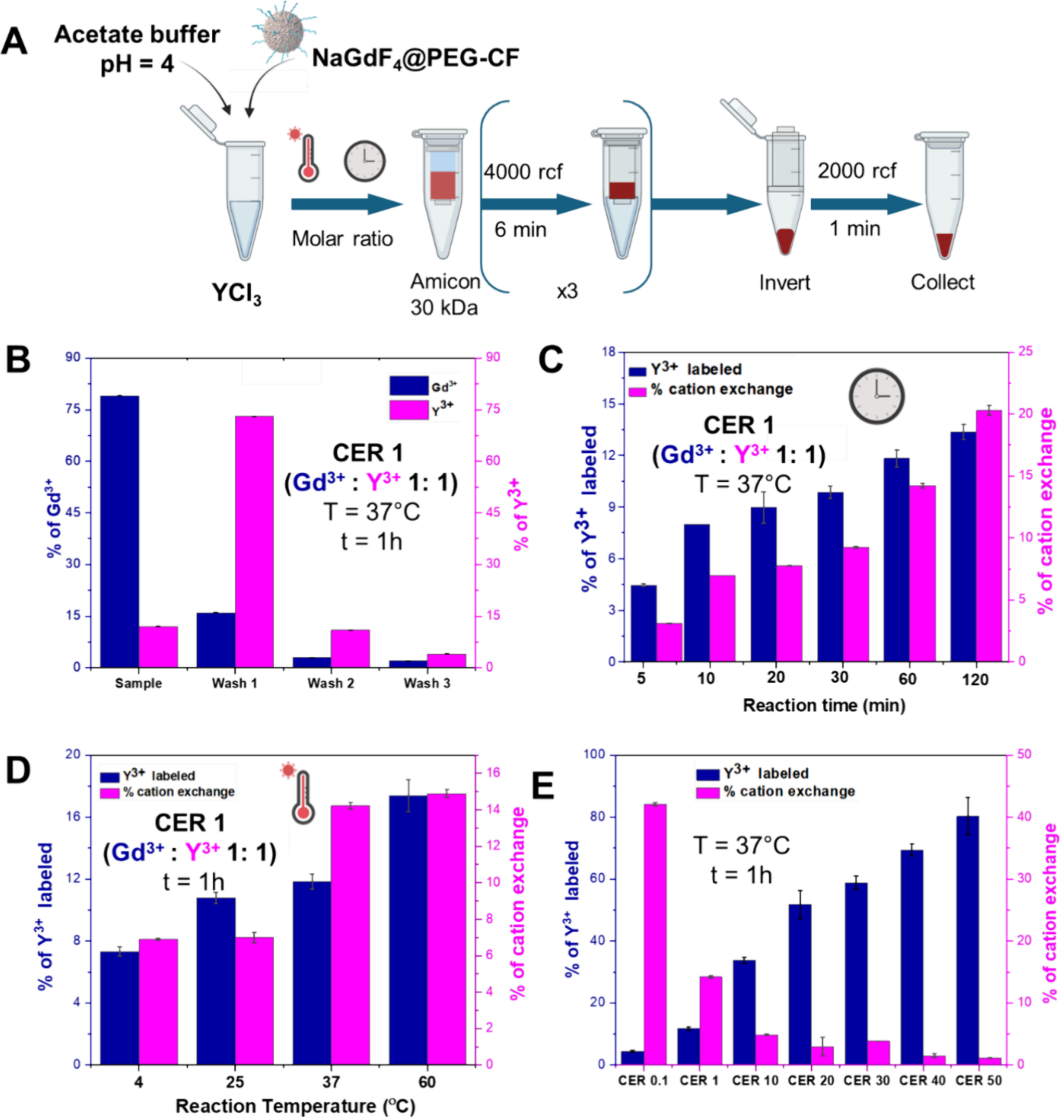
Optimization of the CERs for the insertion of Y^3+^ in NaGdF_4_ NPs. (A) Scheme of the reaction steps, subsequent purification, and isolation of NPs after the exchange. Created with Biorender. (B) CER of Y^3+^ on NaGdF_4_, at a Gd^3+^:Y^3+^ ratio equal to 1 (CER 1), set at 37 °C for 60 min. Elemental analysis of each washing step yields the concentration of incorporated Y^3+^ ions or the exchanged Gd^3+^ ions. Optimization of Y^3+^ insertion by changing the (C) reaction time and (D) reaction temperature. CER 1 was chosen as a representative Gd^3+^:Y^3+^ ratio and to evaluate the difference in labeling yields at different parameters. (E) CER studies of Y^3+^ labeling on NaGdF_4_ NPs at Gd^3+^:Y^3+^ molar ratio of 0.1 (CER 0.1), 1 (CER 1), 10 (CER 10), 20 (CER 20), 30 (CER 30), 40 (CER 40), and 50 (CER 50).

The CER was also optimized with a very similar approach for NaLuF_4_ NPs to obtain NaLuF_4_:Y^3+^(Figure S5). In the case of NaLuF_4_ at a Lu^3+^:Y^3+^ molar ratio of 50:1, the Y^3+^ labeling was ∼78% with a CE percentage of ∼2% per NP (based on the concentration of Lu^3+^ ions) (Figure S5D). Also, for the case of the CE on NaLuF_4_ NPs with Y^3+^ ions, CER was performed at a Lu^3+^:Y^3+^ ratio of 50:1, pH = 4, for 60 min at 37 °C, as optimized previously for NaGdF_4_ and the TEM images of the NaLuF_4_ NPs before and after CER were recorded, indicating that cation-exchanged NPs were stable after CER and did not undergo any visible modification (Figure S6). XRD patterns of NaGdF_4_:Y and NaLuF_4_:Y NPs were also measured after CER at different NPs: Y^3+^ molar ratios, indicating that NPs were stable after CER and did not undergo any phase transition, as evidenced by the consistent diffraction peaks before and after CER, likely due to the low CE percentage (Figure S7). The characteristic peaks for NaGdF_4_ NPs were observed at 28.1, 32.5, 46.5, 55.1, and 75.0° corresponding to (111), (200), (220), (311), and (331), respectively, while the ones of NaLuF_4_ NPs were observed at 28.4, 32.9, 47.3, 56.1, 58.9, 69.1, and 76.3° corresponding to (111), (200), (220), (311), (222), (400), and (331), respectively (Figure S7).

This same successful scheme was used not only for cold CER but also for hot CER with ^90^Y when employing either NaGdF_4_@PEG-CF NPs or NaLuF_4_@PEG-CF NPs.

For the “hot” CE reaction, since the percentage of radiolabeled Y^3+^ associated with the NPs is more important than the percentage of cations exchanged per NP, and because the overall dose determines the radiodose that will be deposited at the tumor, we decided to use the Gd^3+^:Y^3+^ of 50:1 for NaGdF_4_@PEG-CF NPs.

It is worth noting that upon CER with Y^3+^ ions on NaGdF_4_ NPs, Gd^3+^ is released as observed by elemental analysis (Figure 2B wash1, wash2, and wash3). The free Gd^3+^ ions showed faster water exchange, which enhances *T*_1_ contrast useful in MRI with respect to the signal of Gd entrapped into the crystals (Figure 3A). To assess this hypothesis, the *T*_1_ and *T*_2_ relaxation times were measured for the NaGdF_4_@PEG-CF NPs and compared with those times recorded for cation-exchanged NaGdF_4_:Y^3+^ NPs (CER 50), both before and after washing to remove the free Gd^3+^ ions by centrifugation. From the linear fitting of the inverse relaxation time (1/T) versus the concentration of Gd (Figure S8), the slope corresponds to the *R*_1_. The initial R_1_ of NaGdF_4_ was 2.9 mMol^–1^s^–1^, which increased to 4.8 mMol^–1^s^–1^ after 120 min of the CER. This signal increase is likely attributed to the released Gd ions. To check that point, the measurements were repeated after the CE reaction on the NaGdF_4_:Y^3+^ NPs (CER 50) solution and after having washed the sample mixture with Amicon filters, thus removing the released Gd^3+^ ions. The *R*_1_ signal dropped back to 3.09 mMol^–1^s^–1^, indicating the contribution of these free Gd ions to the *T*_1_-enhancement signal during the CE (Figure 3B compares the *R*_1_, *R*_2_, and *R*_1_/*R*_2_ signals of NaGdF_4_@PEG-CF, unwashed NaGdF_4_:Y^3+^ (CER 50), and washed NaGdF_4_:Y^3+^ (CER 50) NPs). For a better understanding of the process, the in situ relaxation times (*T*_1_ and *T*_2_) were measured during the CER (Figure 3C). The results showed a decrease in relaxation times with the reaction time increasing, indicating a continuous release of Gd from the NPs while the reaction proceeded. Furthermore, we performed an MRI on the phantom of NaGdF_4_:Y^3+^ (CER 10) and NaGdF_4_:Y^3+^ (CER 50) along with those of the GdCl_3_ solution (Figure 3D). On NaGdF_4_ NPs, with an increase in reaction time, brightness in MR imaging increased, which also confirmed the exchange of Gd^3+^ in the crystals with Y^3+^ and the Gd release in solution (Figure 3D), thus affecting the longitudinal relaxation time (*T*_1_) during the reaction time. In the case of NaGdF_4_:Y^3+^ (CER 50) NPs, the brightening occurs earlier (already at 20 min) than for the sample NaGdF_4_:Y^3+^ (CER 10) at a lower ratio (Figure 3D and the *T*_1_ value after 120 min was almost the same as the GdCl_3_ solution (Figure 3E).

**Figure 3:**
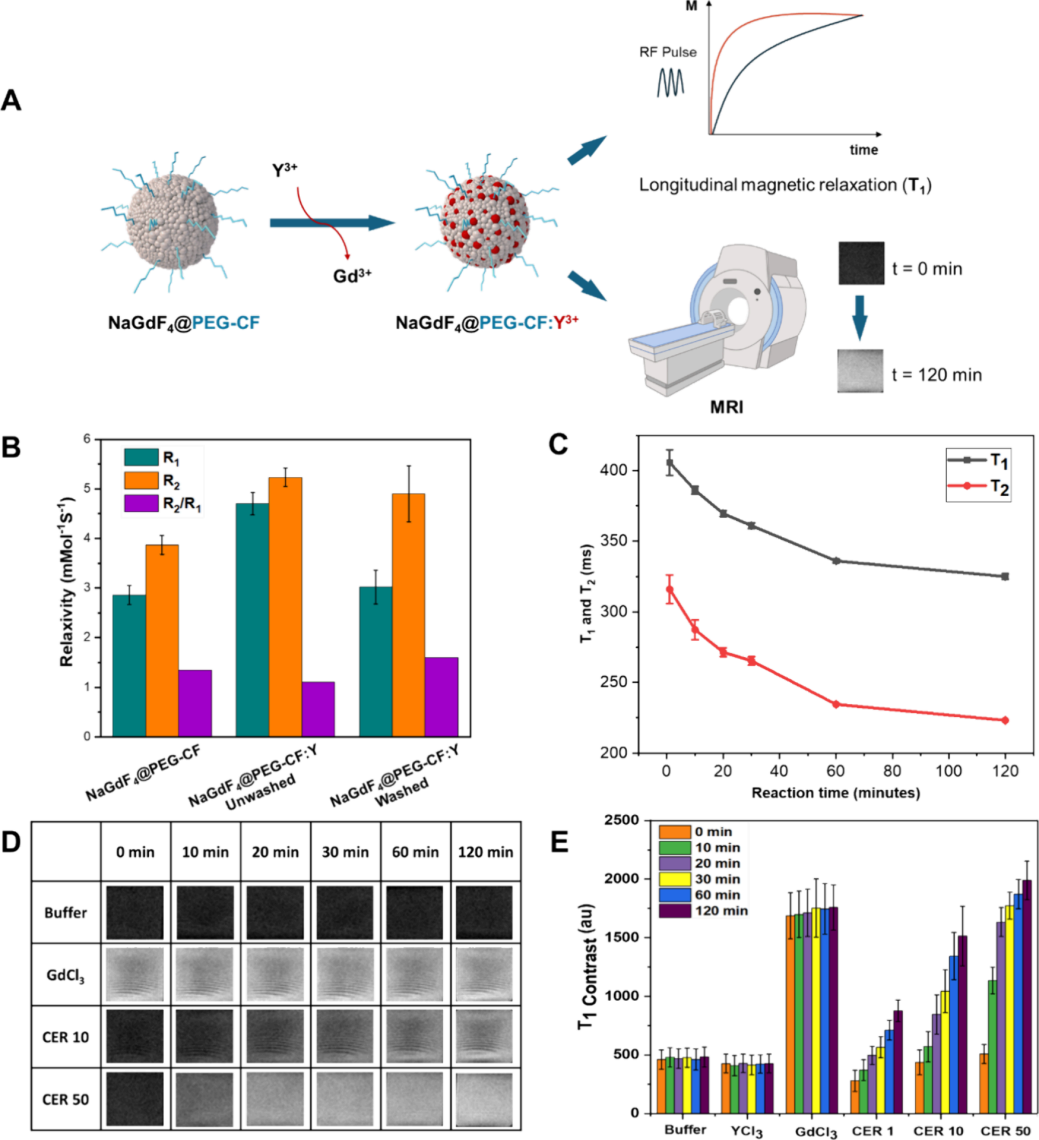
Enhancement of MRI brightness and *T*_1_ signals upon CER on NaGdF_4_@PEG-CF NPs. (A) Scheme of the *T*_1_-weighted measurements and MRI phantom imaging to follow the CE of NaGdF_4_@PEG-CF NPs with Y^3+^ ions. In situ *R*_1_ changes can be measured on a relaxometer, while the contrast enhancement due to the release of Gd^3+^ ions as *T*_1_ signal enhancers can be captured by the use of a three-T MRI machine. Created with Biorender. (B) *R*_1_, *R*_2_, and *R*_2_/*R*_1_ ratios of NaGdF_4_@PEG-CF NPs, unwashed NaGdF_4_:Y^3+^ (CER 50), and washed NaGdF_4_:Y^3+^ (CER 50) (*n* = 3), as measured by 1.5 T relaxometer. (C) In situ changes of *T*_1_ and *T*_2_ relaxation times on NaGdF_4_:Y^3+^ NPs during the CER 50 (*n* = 3), as measured by 1.5 T relaxometer. (D) Phantom imaging at 3T MRI acquisitions over time and corresponding (E) *T*_1_ signal quantification at different Gd:Y ratios (CER 1, CER 10, and CER 50) on NaGdF_4_ NPs along with control samples (YCl_3_, GdCl_3_, and buffer solution) (color code corresponds to different reaction times).

Next, we investigated the insertion of the ^90^Y radioisotope via the same CE method. Based on the “cold” CER protocol, the “hot” reaction of ^90^Y exchange was optimized straight away by choosing to perform the reaction in acetate buffer (pH = 4), at 37 °C, for 60 min and at a Gd^3+^:^90^Y or Lu^3+^:^90^Y ions ratio of 50:1. Briefly, the radiolabeling procedure for the NaGdF_4_ and NaLuF_4_ NPs was performed by mixing ^90^YCl_3_ with 0.5 M sodium acetate buffer at pH = 4 and NaGdF_4_ or NaLuF_4_ nanoparticles (exchange ratio of 50:1) and the resulting reaction mixture was left at 37 °C for 60 min. After the reaction with the NPs, the radiolabeling yield was assessed via radio-TLC, using glass microfiber chromatography paper impregnated with silica gel (iTLC-SG) as the stationary phase and 0.1 M EDTA as the mobile phase. The reaction mixture was further purified by washing with Milli-Q water in an Amicon filter (100 kDa cutoff) (Figure 4A). As shown in Figure 4B, ^90^YCl_3_ migrates to the solvent front on the iTLC, whereas radiolabeled NaLnF_4_:^90^Y remains at the base. The integration of the TLC peaks at the deposition point and at the front of the solvent enabled us to estimate the radiochemical conversion (RCC) that is associated with the NCs. The progress of radiolabeling was monitored with iTLC every 10 min, and it was observed that in the first 10 min, the radiolabeling in NaGdF_4_ was 40% and that in NaLuF_4_ was 30%. This further increased to 73 and 61% over the period of 1 h (Figure 4C). When the reaction was allowed to proceed for an additional hour, the RCC appeared to be slightly decreased; hence, 60 min was chosen as the optimum time point for all further reactions. The purification yield, namely, the radiochemical yield (RCY), was calculated from the final amount of radioactivity associated with the NPs after the reaction and the Amicon filtration step. The RCY revealed that up to 64% NaGdF_4_:^90^Y could be recovered after the radiolabeling, but for NaLuF_4_:^90^Y, the recovery was even lower of about 36% (Figure 4D). This low recovery from amicon filters indicates the higher instability of NaLuF_4_:^90^Y nanoparticle NPs than NaGdF_4_:^90^Y, which may cause an additional loss during the centrifugation step on the filters. The specific activities were calculated to be up to 13 TBq/g for both NPs (refer to Experimental Section for calculations), which are far superior to other specific activities reported on other nanoparticle formulations.^37^ To investigate the NaGdF_4_:^90^Y NPs stability, purified NPs were dissolved in human serum and their long-term stability in terms of colloidal stability and radiolabeling stability was tested. The radiolabeled NPs: ^90^Y, after purification, were incubated with 500 μL of either water or human serum at 37 °C up to 48 h, and the stability was evaluated by radio-TLC in 0.1 M EDTA. It was found that after 48 h, about 72% of NaGdF_4_:^90^Y remains intact in water, whereas for NaLuF_4_:^90^Y, over 66% degraded. In the case of serum stability, both the nanoparticles were deemed unstable, and over 75% and 86% degraded throughout 48 h, respectively (Figure 4E, Figure S9). This would ensure that at least for the first 20 h in serum, the amount of Y-90 on the NPs is quite stable and follows the stability of the NPs.

**Figure 4:**
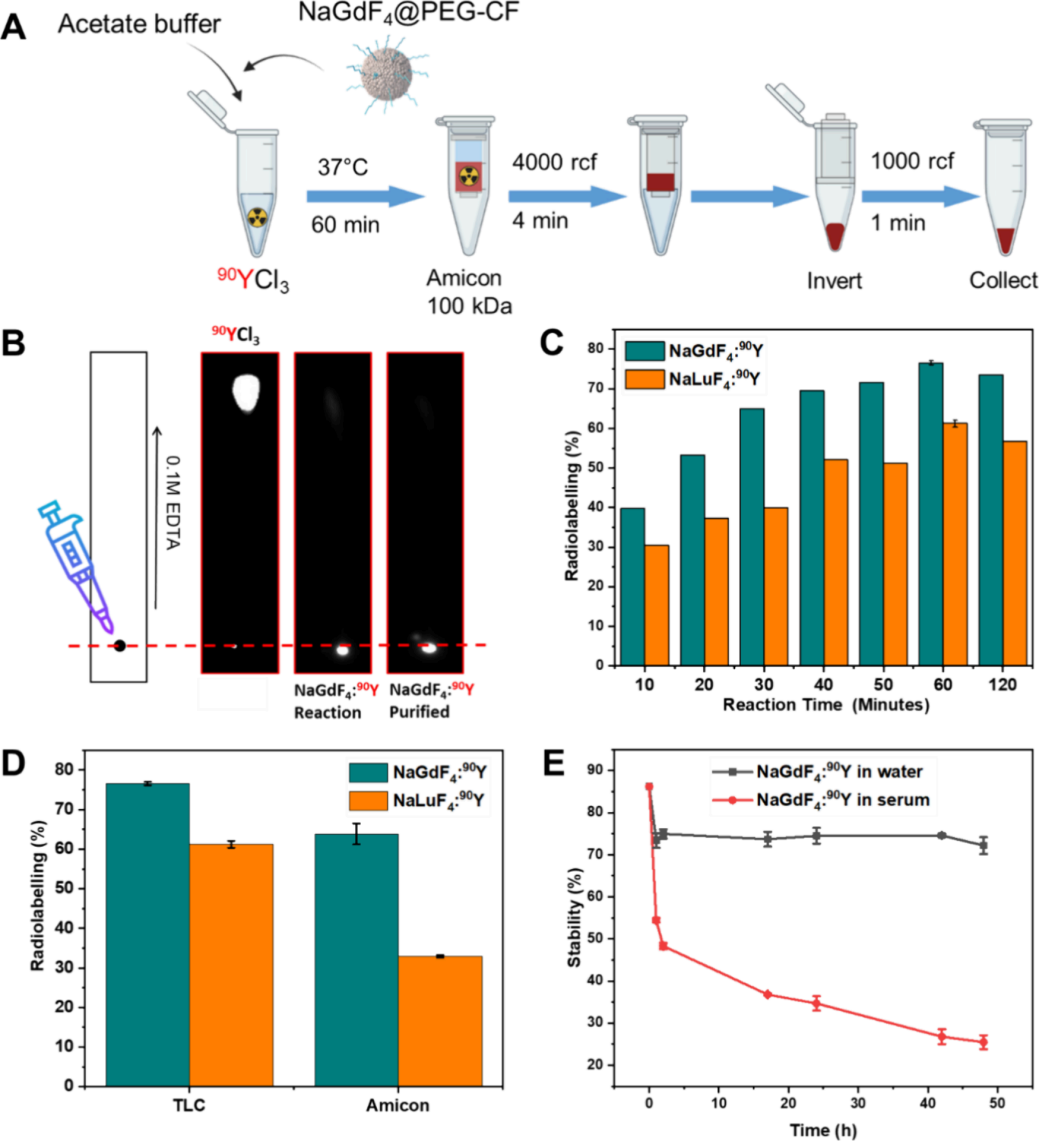
Radiolabeling of NaGdF_4_@PEG-CF NPs with Y-90 (A) Sketch of the radiolabeling protocol via CER for NaGdF_4_@PEG-CF NPs with ^90^YCl_3_ and the subsequent purification and isolation of the exchanged NPs. Created with Biorender. (B) Representative images of the radio-TLC of free ^90^YCl_3_, of NaGdF_4_:^90^Y just after 1 h of CER, and of NaGdF_4_:^90^Y after purification with Amicon filters. (C) Radiolabeling process as a function of time is determined by radio-TLC quantification by measuring the ratio of the amount of radioactive signal at the base of TLC, which corresponds to the NaGdF_4_:^90^Y NP, and to the top of TLC, which represents unlabeled ^90^YCl_3_ (*n* = 3). (D) Radiolabeling yields of two radiolabeling reactions of ^90^Y on NaGdF_4_@PEG-CF and NaLuF_4_@PEG-CF via radio-TLC quantification and purification yields via radioactivity counting using a dose calibrator (*n* = 3). (E) Stability of the NaGdF_4_:^90^Y NPs in water and human serum as determined by radio-TLC quantification (*n* = 3).

Once having identified the best radiolabeling conditions and having verified the stability of the NaGdF_4_:^90^Y NPs, we further assessed the radiotherapy effects of NaLnF_4_:^90^Y NPs (1.85 MBq, which correspond to an amount of 556 or 426 ng NPs, respectively for NaGdF_4_ or NaLuF_4_) were added to the Glioblastoma U-87 cell pellet and incubated for 2 h. Right after the incubation, the cell pellet and NaGdF_4_:^90^Y NPs were plated again into a 6-well plate and supplemented with fresh medium. Cell viability was assessed at 24 and 48 h using Presto Blue (PB) assay (Figure 5A). For NaGdF_4_:^90^Y NPs, after 24 h, there was a strong reduction in cell proliferation (around 50%), which decreased even more after 48 h, reaching almost zero percentage of cells alive (Figure 5B). In the case of NaLuF_4_:^90^Y, cell viability dropped at 20% after 24 h, reaching at 48 h a percentage of 17% (Figure 5C). Worth noting, the nonradiolabeled NPs, along with any components used for the cation exchange reactions (such as the buffer and free metal salts), do not exhibit any cell toxicity on two different cell lines, U-87 glioblastoma and A431 epidermoid carcinoma cells, within the tested concentration range, which for the nonradiolabeled NPs is between 0.05 and 0.25 μM. (Figure 5D,E, Figure S10). These results confirm that both types of NPs are nontoxic when Y-90 free NPs are used at the dose used (0.25 μM, refer to the Gd/Lu concentration) and, when radiolabeled, can be successfully used for radiotherapy, as shown here on glioblastoma tumor cells.

**Figure 5:**
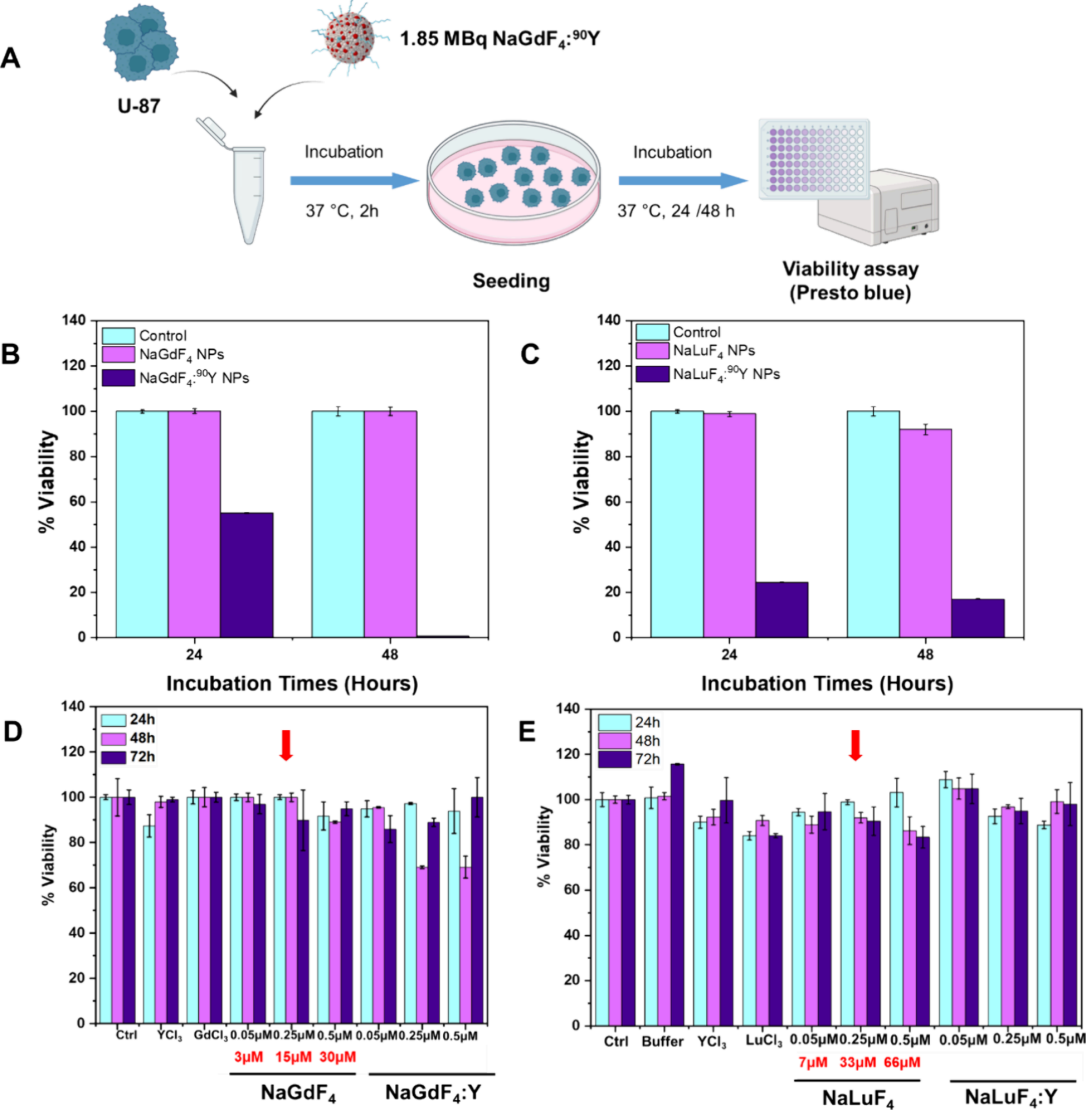
Radiotherapy evaluation of NaGdF_4_@PEG-CF:^90^Y NPs on U-87 cells. (A) Scheme of the in vitro experimental plan to test the radiotherapeutic effect of NaGdF_4_:^90^Y on glioblastoma cells, U-87. U-87 cell pellets (1 × 10^6^ cells) were incubated with NaGdF_4_:^90^Y for 2 h and then further diluted with fresh medium and reseeded on a 6-well plate, and cell viability was assessed at 24 and 48 h by the Presto Blue assay. Created with Biorender. In vitro radiotherapy results of (B) NaGdF_4_:^90^Y and (C) NaLuF_4_:^90^Y on U-87 cells up to 2 days, represented as % of viable cells present (*n* = 3) Biocompatibility tested on the U-87 cells of (D) NaGdF_4_ and of NaGdF_4_:Y, and (E) NaLuF_4_ and NaLuF_4_:Y along with all reagents used in the cation exchange process. Red arrow indicates the concentration of NaGdF_4_ and NaGdF_4_ NPs (based on the concentration of Ln ions as obtained by ICP) used for radiolabeling, whereas the concentration in red corresponds to the concentration of the nanoparticle (calculations as described in the Experimental Section) (*n* = 3).

## Conclusions

This work represents the first successful adaptation of the process of CER for loading pure β-emitting ^90^Y isotopes into rare-earth-based NPs, and it highlights the potential of NaGdF_4_ NPs composition as multifunctional platforms for radiotherapy and MRI imaging of the exchange process. By initially using nonradioactive Y^3+^, we have first established the optimal parameters—such as temperature, reaction time, and ion ratios—to optimize the cation exchange, and we laid the basis for safe and effective translation of the protocol to the radiolabeling process when using ^90^Y ions. In the case of NaGdF_4_ NPs, the release of Gd^3+^ during the exchange served as a built-in marker for monitoring the exchange reaction by T_1_ MRI signal increase. At the same time, this experiment demonstrated the use of MRI in monitoring cation exchange on NaGdF_4_ NPs in situ. As a next step, it may be possible to monitor CER occurring on NaGdF_4_ NPs injected directly at the tumor with a radioisotope injected systemically, thus evaluating, by MRI signal change, the radionuclide sequestering ability of NaGdF_4_ NPs, in situ, in a tumor microenvironment. The cytotoxicity of radiolabeled NaLnF_4_:^90^Y NPs tested on U-87 glioblastoma cells is here providing the proof of concept of the use of ^90^Y radiolabeled NPs obtained by CER, in an internal radiotherapy study, even when employed at low radiodose levels (1.85 MBq). These results underscore the therapeutic potential of radiolabeled NPs obtained by the CER approach.

Future material research studies will aim at implementing the same cation exchange radiolabeling strategy to other therapeutically relevant isotopes such as ^177^Lu and ^212^Bi ions, advancing the clinical utility of the resulting radiolabeled upconverting NPs. ^177^Lu radionuclide will provide longer radio decay half-life (6.7 days) and medium-range β^–^ emissions ideal for treating larger tumor masses, while ^212^Bi ions have potent α-emissions, which will make it highly effective for eradicating small, resistant tumor sites or micro metastases.^19,48–50^

For doable clinical translation, we should also prioritize surface engineering studies on the NPs to enhance their colloidal and radiolabeling stability in biological environments, aiming at systemic administration and the specific target of tumor cells. Between the two compositions in the test tube study, NaGdF_4_ showed better colloidal and radiolabeling stability than NaLuF_4_ NPs. In vivo validation using murine tumor models will also be critical to assess biodistribution, therapeutic efficacy, stability, and safety under physiologically relevant conditions, facilitating the transition of these nanoplatforms from proof-of-concept to real clinical applications.

## Experimental Section

### Materials

Ethyl 2-bromophenylacetate (EtBPhAc, 97%, CAS no. 2882–19–1), *N*,*N*,*N*′,*N*″,*N*′′-pentamethyldiethylenetriamine (PMDETA, 99%, CAS no. 3030–47–5) and copper­(II) bromide (CuBr_2_, 99%, CAS no. 7789–45–9), sodium trifluoroacetate (Na­(tfa), 98%, CAS no. 2923–18–4), trifluoroacetic acid (tfa, 98%, CAS no. 76–05–1), yttrium­(III) chloride hexahydrate (YCl_3_.6H_2_O, 99.99%, CAS no. 10025–94–2), gadolinium­(III) chloride hexahydrate (GdCl_3_.6H_2_O, 99.99%, CAS no. 13450–84–5), lutetium­(III) oxide (Lu_2_O_3_, 99.999% CAS no. 12032–20–1), furfurilamine (FA, 99.9%, CAS no. 617–89–0), poly­(ethylene glycol) methyl ether methacrylate (PEGMA, Mw: 950 g·mol^–1^, 95%, CAS no. 26915–72–0), *N*-hydroxy succinimide (NHS, 98%, CAS no. 6066–82–6), methacryloyl chloride (97%, CAS no. 523216), dopamine hydrochloride (DOPA.HCl, 98%, CAS no. 62–31–7), triethylamine (TEA, > 99%, CAS no. 121–44–8), oleic acid (OA, 90% CAS no. 112–80–1), oleyamine (OLA, 70%, CAS no. 112–90–3), 1-octadecene (OD, 90%, CAS no. 112–88–9), ethylenediaminetetraacetic acid disodium salt dihydrate (EDTA, 99%, CAS no. 6381–92–6), and sodium acetate (≥99%, CAS no. 127–09–3) were purchased from Sigma-Aldrich and used as received without any further purification. Gadolinium­(III) oxide (Gd_2_O_3_, 99.999%, CAS no. 12064–62–9) was purchased from Strem Chemicals. *N*-Succinimidyl methacrylate (NSMA) was synthesized by the esterification between NHS and methacryloyl chloride as described by us in the literature.^46^ All of the solvents were purchased from commercial sources such as Sigma-Aldrich with the highest available purity (not lower than 95%), and they were used as received. ^90^YCl_3_ (1 mCi, radiochemical purity > 95%) was purchased from PerkinElmer, USA.

### Characterization

The particle sizes were characterized by dynamic light scattering (DLS) using a Malvern Instruments Zetasizer nano series instrument. An equilibration time of 1 min was allowed prior to each reading, and at least three replicate measurements were made for each sample. Transmission electron microscopy (TEM) images were obtained using a JEOL JEM 1400 electron microscope, equipped with a W thermionic electron source and an 11 MP Orius CCD camera (Gatan company, USA), with an acceleration voltage of 100 kV. The samples were prepared by drying a drop of the sample onto a carbon-coated copper grid, which was then left to dry before imaging.

Elemental analysis was conducted via Inductively Coupled Plasma (ICP) Atomic Emission Spectroscopy on a ThermoFisher CAP 6000 series. The samples from cation exchange reactions were prepared by digesting 10 μL of the sample in 1 mL of aqua regia (concentrated HCl:HNO_3_ 3:1) overnight, followed by dilution with Milli-Q water to 10 mL. For all the other samples that do not refer to the cation exchange, the 10 μL samples were digested in the same way but were digested in 2.5 mL of aqua regia and diluted to 25 mL of Milli-Q water.

Fourier transform infrared (FTIR) spectra were measured on a Bruker Vertex 70v Fourier transform infrared spectrometer. The spectra were collected over the wavenumber range of 400–4500 cm^–1^ with a resolution of 4 cm^–1^, and 64 scans were accumulated to minimize spectral noise.

Powder X-ray diffraction (XRD) patterns were obtained using a PANalytical Empyrean X-ray diffractometer equipped with a 1.8 kW Cu Kα ceramic X-ray tube and a PIXcel3D 2 × 2 area detector operating at 45 kV and 40 mA. The diffraction patterns were collected in air at room temperature by using parallel-beam geometry and symmetric reflection mode. All XRD samples were prepared by drop-casting a concentrated solution on a zero-diffraction quartz wafer.

### Synthesis of the Monomer NHS Methacrylate (NHSMA)

This protocol was reported in our previous publication.^43,46^ To summarize, in a 500 mL flask, *N*-hydrosuccinimide (25 g, 0.22 mol) was dissolved in 300 mL of chloroform. Then trimethylamine (34.5 mL, 0.25 mol) was added, and the reaction was cooled to 0 °C. Methacryloyl chloride (23.4 mL, 0.24 mol) was added dropwise to the stirred reaction mixture over a 15 min period. After being stirred for an additional 60 min at 0 °C, the solution was washed with 160 mL of ice-cold water and 160 mL of saturated brine (concentrated solution of sodium chloride in water). Then the solution was dried with MgSO_4_, filtered, and concentrated in vacuo using a rotary evaporator to a volume of 60 mL. Cold hexane was added to precipitate the solution, and then it was filtered using a Buchner flask setup. This step was performed one more time, using ethyl acetate for the dissolution and hexane for the recrystallization. Finally, the white crystal was collected and dried in a vacuum oven at 30 °C (32.14 g, 0.17 mol, 76%). The NHSMA powder was kept at −20 °C.

### Synthesis of the Copolymer Poly­(PEGMA-co-NHSMA)

This protocol was reported in our previous publication.^46^ To summarize, PEGMA (7.70 g, 0.02 mol) was weighed under air and stored in the refrigerator at −20 °C until the next day. Then the PEGMA monomer was dissolved in 8 mL of DMF and left in the orbital shaker at a very high speed for 4 h. After this time, NHSMA (2.18 g, 0.01 mol) was added to the solution and stirred for a further 15 min to completely dissolve the monomers. Subsequently, EtPBrAc (175 μL) and PMDETA (26.9 μL) were added to the solution, sealed with a cap with a septum, and purged with N_2_ flow for 10 min. In the meantime, a CuBr solution was prepared in DMF (3.2 mg/mL, in 3 mL of DMF), and purged with N_2_ flow for 10 min. After this time, 3 mL of the copper catalyst solution was injected in one shot into the solution containing the monomers, ligand, and initiator, stirred for 5 min under N_2_, and left under a UV lamp in the cold room for 18 h to initiate the polymerization. The UV source used was a nail curing lamp (λ_max_ = 360 nm) equipped with four 9W bulbs. Then, the reaction was stopped and dissolved in Acetone (10 times more than the volume of the solution, which means roughly 30 mL). Next, the reaction mixture of the polymer was recovered by a column (diameter of 3 cm and length of 30 cm) filled with basic alumina and concentrated, reaching ∼20 mL with the rotavap. The polymer was then precipitated by adding cold Diethyl Ether, centrifuged for 10 min at 2500 rpm, and dried in a vacuum oven at 30 °C (7.80 g, 77%).

### Synthesis of the PEG-CF-Based Polymer

This protocol was reported in our previous publication.^43^ To summarize, the Poly-(PEGMA-co-NHSMA) polymer (1.62 g) was dissolved in 8.08 mL of DMF in order to reach a concentration of 0.2 mg/mL, in a vial equipped with a magnetic stirrer. Then, DOPA HCl (316 mg), furfurylamine (205 μL), and TEA (302 μL) were added. Furfurylamine is used to reduce hydrophobicity, and TEA is used to neutralize HCl and accelerate the aminolysis. After 24 h, the reaction product was purified by dialysis using a cellulose membrane with a molecular weight cutoff of 100 kDa against 2 L of HCl solution (0.01M) for 48 h to remove the unreacted residues. Then, the product was filtered using RC Santorus filters (0.45 μm) and frozen, dried, recovered, and stored (1.39 g, 86%).

### Synthesis of the NaLuF_4_ Nanoparticles

This protocol was replicated based on a previously reported procedure in the literature.^39^ Briefly, in a 50 mL three-neck flask were added Lu_2_O_3_ (298.4 mg, 0.75 mmol), 4 mL of tfa, and 4 mL of H_2_O, together with a magnetic stirrer. After dissolution under stirring, the solution was completely transparent, and the solvents slowly evaporated in order to obtain the solid lutetium precursor, Lu­(tfa)_3_. After the precursor was dry, in the same flask, Na­(tfa) (206.6 mg, 1.5 mmol), 5.3 mL of oleic acid (OA), 10.7 mL of octadecene (OD), and 7.05 mL of oleylamine (OM) were added and let under vacuum at 100 °C for 30 min to dissolve the precursors. Then, the reaction mixture was left under N_2_ for 10 min at 310 °C to form the nanoparticles. The reaction suspension was dissolved in 20 mL of EtOH and centrifuged at 5000 rpm for 5 min on a Sigma 3–16P centrifuge. The pellet was then dissolved in 20 mL of EtOH, sonicated, and centrifuged at 5000 rpm for 5 min. After that, 10 mL of EtOH was added to the pellet, and the solution was sonicated. If the dispersion of the particles was difficult, 10 mL of water was added and stirred for some minutes. This addition of water was required to remove the NaF that is formed during the reaction as a whitish precipitate. Then the solution was centrifuged at 5000 rpm for 5 min. The pellet was again dispersed in 20 mL of EtOH, sonicated, and centrifuged under the same conditions. At the end, the pellet was dispersed in 20 mL of CHCl_3_ and sonicated for a few minutes. A transparent solution was observed.

### Synthesis of the NaGdF_4_ Nanoparticles

This protocol was reported previously in the literature^39^ and reproduced by us with minor modifications. Briefly, in a 50 mL three-neck flask were added Gd_2_O_3_ (273.7 mg, 0.75 mmol), 4 mL of tfa, and 4 mL of H_2_O, together with a magnetic stirrer. After dissolution, the solution was completely transparent, and the solvent was slowly evaporated to obtain the solid precursors, Gd­(tfa)_3_. After the precursors were dry, Na­(tfa) (274.6 mg, 2 mmol), 5.3 mL of OA, 10.7 mL of OD, and 7.05 mL of OM were added and left under vacuum at 100 °C for 30 min to dissolve the precursors. Then, the reaction was run under N_2_ for 10 min at 310 °C to form the nanoparticles. The reaction suspension was dissolved in 20 mL of EtOH and centrifuged at 5000 rpm for 5 min using the same centrifuge mentioned above. The pellet was then dissolved in 20 mL of EtOH, sonicated, and centrifuged at 5000 rpm for 5 min. After that, 10 mL of EtOH was added to the pellet, and the solution was sonicated. If the dispersion of the particles was difficult, then 10 mL of water was added and stirred for some minutes. This addition of water was also necessary to eliminate the NaF formed during the reaction as described above. Then, the solution was centrifuged at 5000 rpm for 5 min. The pellet was dispersed in 20 mL of EtOH, sonicated, and centrifuged under the same conditions. At the end, the pellet was dispersed in 20 mL of CHCl_3_ and sonicated for a few minutes. A transparent solution was observed and kept in a 10 mL vial in a cold room.

### Ligand Exchange on NaLnF_4_ Nanoparticles with PEG-CF-Based Polymer

This protocol was reported in our previous publication for other types of NPs and here adapted for NaLnF_4_ NPs.^43^ In an 8 mL vial, 1 mL of the polymer stock solution (PEG-CF-based polymer dissolved in CHCl_3_, 100 mg/mL) and NaLnF_4_ NPs solution (5 mg, 5.225 mg/mL) were added. After sonication, 25 μL of TEA was added, and the solution was left on a Heidolph orbital shaker (1500 rpm) at RT for 18 h. The reaction suspension was precipitated using hexane and centrifuged for 10 min at 2000 rpm. The pellet was dissolved in 1 mL of THF, again precipitated using hexane, and then centrifuged for 10 min at 2000 rpm. After that, the pellet was dried and transferred to 1 mL of H_2_O with sonication. The product was washed and concentrated 3 times with H_2_O using an Amicon 100 kDa and centrifuging at 2000 rpm for 20 min each time. Finally, the nanoparticles were dispersed in 500 μL of H_2_O and characterized.

### Cation Exchange Reactions (CERs)

In a typical CER, in a 1.5 mL Eppendorf vial containing 150 μL of sodium acetate buffer (0.3 M, pH = 4), 53.2 μL of YCl_3_ (pH = 4, 0.094 M, 5 μmol) was added and after vigorously mixing 260 μL of NaLnF_4_ NP (0.193 M, 50 μmol in NP) was added ths reaching a relative amount of Ln^3+^(NP):Y^3+^ of 10:1. The CER was left taking place for 10 min at 37 °C. The cation exchange ion ratios, time, and temperature were changed as needed for optimization. After the prefixed reaction time, the reaction mixture was transferred into a 0.5 mL 30 kDa MWCO Amicon centrifugal filter (Merck), filled with H_2_O up to 0.4 mL total volume, and centrifuged at 4000 rcf for 6 min such that the solution volume of the filter was reduced to approximately 100 μL. This step was repeated two more times, refilling, each time, the cartridge with 400 μL of H_2_O. The washing solutions and the purified NP, the latter collected on top of the filter with a rough sample volume of 80 μL, were then analyzed by ICP-OES.

### Relaxometry

Relaxivity measurements were performed using a water solution of NaGdF_4_ with gadolinium (Gd) concentration ranging from 0.005 to 1 mM. The longitudinal (*T*_1_) and transverse (*T*_2_) relaxation times were measured at 40 °C using a Minispec spectrometer (Bruker, Germany) mq 60 (1.5 T). The *T*_1_ relaxation profile was obtained using an inversion–recovery sequence, with 60 data points and three acquisitions for each measurement. In order to study the in situ changes of *T*_1_ and *T*_2_ relaxation times on NaGdF_4_:Y^3+^ NPs during CER 50, the exchange was carried out as described above. Relaxivity measurements were acquired as soon as the reaction was completed, after 60 min of incubation, and then after passing the reaction solution through the Amicon filters.

### MRI Imaging

To investigate the contrast enhancement due to the in situ release of Gd^3+^ during the CER, MR phantom studies were performed using a 3T small animal MRI scanner (MRS*PET 7.0 T, MR Solutions, Guildford, UK). In an 8-well chambered coverslip (Ibidi GmbH, Germany), 400 μL of the following samples: (1) 0.5 M sodium acetate buffer, (2) YCl_3_, (3) GdCl_3_ solution, (4) NaGdF_4_ NPs only, and (5) NaGdF_4_ NPs + YCl_3_ (calculated according to the CER ratio that needs to be achieved) were added. The MR images were obtained using *T*_1_ weighted Fast Spin Echo (FSE-T_1_) sequence with the following parameters set on the instrument: coronal mode, echo times (TE) = 11 ms, repetition times (TR) = 1000 ms, field of view (FOV) = 60 × 60 mm, flip angle 90°, slice thickness = 0.5 mm, and average = 3.

### Radiolabeling

In a 1.5 mL Eppendorf vial, a variable quantity (10–20 μL) of ^90^YCl_3_ corresponding to a gross activity of 9.25 MBq (0.25 mCi), 60 μL of 0.5 M sodium acetate buffer (pH = 4) and NaLnF_4_ NP solution (containing in Ln equivalent 50 times that of ^90^YCl_3_ to keep a 50:1 ratio) were added. The reaction mixture was incubated at 37 °C. Every 10 min, 1 μL of the reaction mixture was spotted on iTLC-SG chromatography paper (Agilent Technology), and the iTLC was developed in 0.1 M EDTA (pH = 7.5) aqueous solution as the mobile phase. The dried iTLC plates were exposed to a photographic impressing plate for 30 s, and the film was analyzed on an appropriate densitometry imaging scanner system (FLA-9000, PerkinElmer). The radiochemical conversion (RCC) based on TLC was calculated from the ratio of % of signal found on the deposition point to the % of signal found on the solvent front corresponding to the free ^90^YCl_3_ migration.

### Concentration and Purification of the Solution of Radiolabeled Nanocrystals

The washing and concentration of the nanocrystals after the radiolabeling procedure were performed using 0.5 mL of 100 kDa MWCO Amicon centrifugal filters (Merck). After the CER, the reaction mixture (approximately 80 μL) was transferred into an Amicon filter inserted in an Eppendorf vial, and Milli-Q water was added up to 500 μL. After 4 min of centrifugation at 4000 rcf, the filter containing radiolabeled NaLnF_4_ was removed and inserted upside down in a new vial and centrifuged for 1 min at 1000 rcf. Using a dose calibrator (VDC-603, Comecer), we checked the activity flow at each step of the purification was checked. The radiochemical yield (RCY) was calculated as the percentage of activity recovered with respect to the starting activity.

### Stability Tests of the Radiolabeled Nanocrystals

Purified NaLnF_4_:^90^Y NPs were tested for their long-term stability in Milli-Q water as well as human serum. The radiolabeled NaLnF_4_ NPs (740 kBq or 20 μCi) in 10 μL were incubated with 50 μL of Milli-Q water or human serum at 37 °C up to 48 h. One μL of sample was extracted from the reaction vials at desired time points (in triplicate) and spotted on iTLC. The iTLC was developed in 0.1 M EDTA (pH = 7.5) as mobile phase and imaged as described above.

### Calculations of Specific Activity

The mass of the NPs used for radiolabeling was calculated following eq 1

$$\mathrm{mass}=\mathrm{concentration}\;(\frac{\mathrm{mol}}{L})\times \mathrm{volume}\;(L)\times \mathrm{molecular}\;\mathrm{weight}\;\left(\frac{g}{\mathrm{mol}}\right)$$ 1

These masses of NPs were found to be 5.56 × 10^–7^ g for NaGdF_4_ and 4.26 × 10^–7^ g for NaLuF_4_, considering a molecular weight of 380429.045 and 1262603.023 per NPs, respectively, obtained by density from XRD analysis, volume of NCs from TEM measurements, and the elemental concentration from ICP.

The specific activity of the radiolabeled NaLnF_4_ was then calculated taking into account the mass of nanoparticles (as determined by eq 2) that was used in the radiolabeling experiments and the initial activity and radiochemical conversion (RCC) obtained by TLC analysis of the crude reaction.

$$\mathrm{specific}\;\mathrm{activity}\;(\mathrm{TBq}/g)=\frac{\mathrm{activity}\;(\mathrm{TBq})}{\mathrm{mass}\;\mathrm{of}\;\mathrm{NCs}\;(g)}\times \mathrm{RCY}$$ 2

For an average reaction of 9.25 MBq and RCC of 75 and 60% for NaGdF_4_ and NaLuF_4_, specific activities were found to be 13.2 and 13 TBq/g, respectively.

### Cell Culture

U-87 glioblastoma cancer cells (ATCC HTB-14) and A431 human squamous carcinoma cells (ATCC CRL-1555) were cultured in Dulbecco’s modified medium (DMEM) supplemented with 10% fetal bovine serum (FBS), 1% pennicillin streptomycin (PS), and 1% glutamine and cultured at 37 °C, 5% CO_2_, and 95% humidity. Cells were split every 3–4 days, before reaching 75% confluence. The radiotoxicity experiments were performed at passage between P8 and P10.

### Cell Study

#### Biocompatibility Test

Before testing the therapeutic effect on the ^90^Y-labeled NPs, the biocompatibility of NaGdF_4_ and NaLuF_4_ NPs before and after CER with cold YCl_3_ was first tested. For the cytotoxicity experiment, 1 × 10^6^ cells of the U-87 or A431 cell line were plated and cultured in a 6-well plate in order to reach 75% confluence the day after seeding. Different concentrations of nanoparticles (0.05 μM, 0.25 μM, and 0.5 μM based on the Gd or Lu concentration as obtained by ICP) for both pristine NaGdF_4_ and NaLuF_4_ NPs and those that undergo CER with cold yttrium (YCl_3_), were tested: In addition to these samples, other controls were added including a sample made of nontreated cells which did not undergo any treatment (Ctrl) but cultured under the same conditions of the treated samples, a sample of cells exposed to 0.5 M sodium acetate buffer at pH 4 which is the buffer of the reaction (named buffer); a sample of cells exposed to cold yttrium (named YCl_3_) and a cell sample exposed to GdCl_3_ (named GdCl_3_). To note that all samples (NPs or salt solutions) prior to being added to the cells were exposed to a 20 min UV light (λ = 254 nm) under a biological hood. Fresh medium was then added directly to the NP/salt solution vial, and the medium was pipetted to homogenize each sample. Upon removal of the medium from the well, 0.5 mL of new medium (containing NPs/Salts at the concentration above specified) was added per well. Cells were then incubated at 37 °C in a cell culture incubator. The viability was evaluated by measuring the cell metabolic activity by means of Presto Blue assay at 24, 48, and 72 h after continuous exposure.

#### Therapeutic Effect

To test the radiotherapeutic effect of radioactive NaGdF_4_:^90^Y and NaLuF_4_:^90^Y after CER, only the U-87 cell line was used. Three conditions were tested: a sample of nontreated cells considered as the negative control (Ctrl), and samples of cells incubated with radiolabeled nanoparticles, either NaGdF_4_:^90^Y NPs or NaLuF_4_:^90^YNPs. The day before the CER with ^90^Y, 1 × 10^6^ cells/well were plated into a 6-well plate. For the exposure to the Y-90 labeled NPs, upon detaching the cells using 500 μL of Trypsin EDTA/well and centrifuging at 1000 rpm for 5 min, the supernatant was discarded, and 1.85 MBq (50 μCi) of radiolabeled medium, diluted into 100 μL of medium, was added to the corresponding pellet sample, without breaking the cell pellet. The samples were incubated at 37 °C in a cell culture incubator for 2 h. After this period, the cell pellet mixed with the NPs was diluted with 1 mL of cell medium and replated into a 6-well plate. The viability of the cells was measured by means of Presto Blue assay at 24 and 48 h postcontinuous exposure.

## Supplementary Material



## Notes

Comparative features of ^90^Y/^177^Lu/^124^I- -radiolabeled rare-earth NPs (Table S1), crystallographic XRD data (Table S2) and FTIR spectral peaks of the UCNPs (Table S3), further morphological and structural characterization of the UCNPs, including TEM images and particle size analysis (Figures S1 and S2) XRD patterns of synthesized materials (Figure S3), DLS of the water-transferred materials (Figure S4), optimization study for cation exchange reactions on NaLuF_4_ NPs (Figure S5), additional TEM images of the NaLuF_4_ and NaLuF_4_ radiolabeled with Y (Figure S6), XRD patterns for NaGdF_4_ NPs and NaLuF_4_ NPs before and after the CERs with Y (Figure S7), calibration curves for *T*_1_ relaxivity measurements (Figure S8), colloidal stability study of NaLuF_4_:^90^Y in water and human serum (Figure S9), and biocompatibility assay results of NaGdF_4_, NaLuF_4_, NaGdF_4_:Y, and NaLuF_4_:Y on the A431 cell line (Figure S10) (PDF)
