# Supplementary material for: Cation Exchange Protocol to Radiolabel Rare-Earth Nanoparticles with Yttrium-90 for Radiotherapy and for Magnetic Resonance Imaging
Source: ACS Appl Mater Interfaces. 2025 Jun 9;17(24):35181–94. doi: 10.1021/acsami.5c05495 (PMC12200231; doi:10.1021/acsami.5c05495)
Supplement: Supplementary file 1 [file am5c05495_si_001.pdf]

SUPPORTING INFORMATION

Cation exchange protocol to radiolabel rare-earth nanoparticles with Yttrium-90 for radiotherapy and for MRI imaging

Nisarg Soni[&], Ana Maria Panaite[&], Tuhin Samanta[&], Giulia E.P. Nucci[&], Emille M. Rodrigues[&] and Teresa Pellegrino[&]*

[&] Italian Institute of Technology, via Morego 30 16163, Genoa

[*] Correspondence to Teresa.Pellegrino@iit.it

Table S1: Comparative overview properties of radiolabeled rare-earth NPs incorporating ^{90}Y or ^{77}Lu, or ^{124}I radionuclides

Parameters	^{90}Y-Rare-Earth NPs	Other Radiolabeled Rare-Earth Nanoparticles (e.g., ^{177}Lu-Rare Earth NPs, ^{124}I-Rare Earth NPs)
**Decay mode**	Pure β- emitter.[1]	^{177}Lu β^- and γ emitter[2]<br>^{124}I β^+ and γ emitter[2]
**Energy levels**	0.93 MeV (ideal for tumor penetration).[1]	^{177}Lu (β^-: 0.498 MeV γ:113 keV)[2]<br>^{124}I (β^+: 1.53 MeV; γ: 603 keV)[2]
**Half-life**	2.67 days.[1]	^{177}Lu: 6.7 days<br>^{124}I: 4.18 days[2]
**Radiotoxicity & Safety**	Relatively low external radiation, minimal side damage.[1]	Both may involve higher gamma radiation, with potential off-target exposure.[2]
**Therapeutic efficacy**	High, with proven success in multiple cancer models.[3,4]	Often effective, but may require higher doses and longer exposure.[5,6,7]

**Therapeutic dose**	High-energy beta particles allow lower administered activities to achieve therapeutic outcomes, reducing systemic exposure.[3,4,6]	Lower-energy beta emitters may need higher injected doses for similar efficacy.[5,6,7]
**Clinical translation***	^{90}Y radionuclide is FDA-approved, widely used in clinical radiotherapy (e.g., SIRT), with established dosimetry and regulatory pathways.[8,9]	^{124}I radionuclide is not yet FDA-approved as PET radiotracer[10]<br>^{177}Lu radionuclide is FDA approved for radiotherapy[11]

***None of the radiolabeled Rare-Earth NPs is clinically approved**

Table S2: Summary data of X-Ray diffraction. D-spacing calculated using Bragg's Law Equation (Equation S1).

Sample	2θ (°)	d-spacing (Å)	Phase	Crystal Plane (hkl)
**$NaGdF_4$**	28.1	3.17	Cubic-fluorite (CaF_2)	(111)
	32.5	2.75	Cubic-fluorite (CaF_2)	(200)
	46.5	1.95	Cubic-fluorite (CaF_2)	(220)
	55.1	1.67	Cubic-fluorite (CaF_2)	(311)
	75	1.27	Cubic-fluorite (CaF_2)	(331)
**$NaLuF_4$**	28.4	3.14	Cubic-fluorite (CaF_2)	(111)
	32.9	2.72	Cubic-fluorite (CaF_2)	(200)
	47.3	1.92	Cubic-fluorite (CaF_2)	(220)
	56.1	1.64	Cubic-fluorite (CaF_2)	(311)
	58.9	1.57	Cubic-fluorite (CaF_2)	(222)
	69.1	1.36	Cubic-fluorite (CaF_2)	(400)
	76.3	1.25	Cubic-fluorite (CaF_2)	(331)

Bragg's Law equation:

$$n\lambda = 2d\sin\theta \qquad \text{Equation S1}$$

Where n is the order of diffraction, λ is the wavelength of the incident radiation, d is the distance between the crystal planes, and θ is the angle of incidence.

Table S3: Summary data of FT-IR.

Wavenumber (cm^{-1})	Vibration type	Assignment	Observed in
**3500**	Stretching	N–H stretching	PEG-CF
**3300**	Stretching	O–H stretching	PEG-CF
**2943**	Asymmetric CH_3 stretching (ν_as)	Terminal methyl group	PEG-CF
**2934**	Asymmetric CH_3 stretching (ν_as)	Terminal methyl group	Oleylamine, Pristine $NaGdF_4$ NPs
**2885**	Asymmetric CH_2 stretching (ν_as)	Methylene group	PEG-CF
**2840**	Asymmetric CH_2 stretching (ν_as)	Methylene group	Oleylamine, Pristine $NaGdF_4$ NPs
**1700**	Stretching (ν_s)	C=O (ester)	PEG-CF
**1600**	Stretching (ν_s)	C=O (amide)	PEG-CF
**1580**	NH_2 scissoring + N–H bending	Amine group (from oleylamine)	Oleylamine, Pristine $NaGdF_4$ NPs

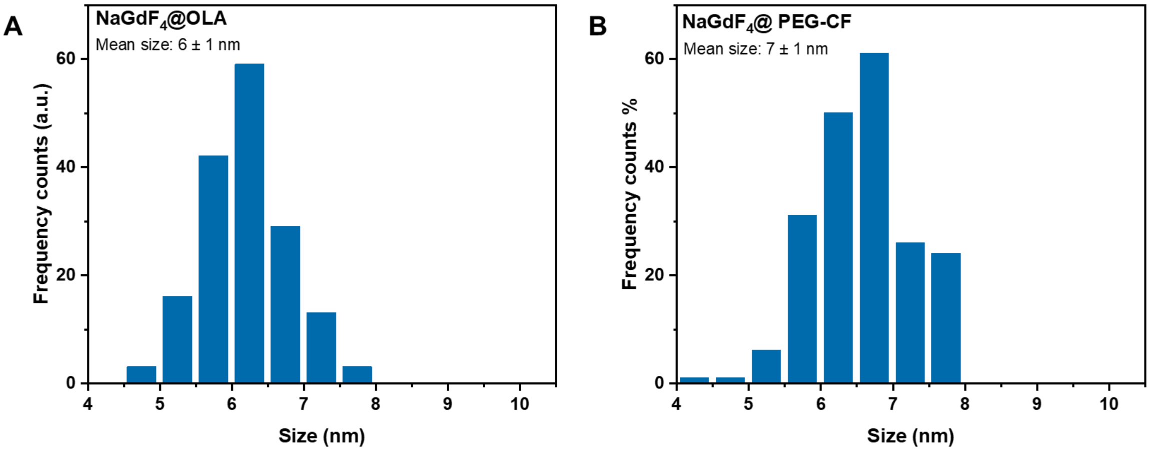

Figure S1. Size distribution histograms of $NaGdF_4$@OLA NPs (A) and $NaGdF_4$@PEG-CF NPs (B) as measured from their TEM images.

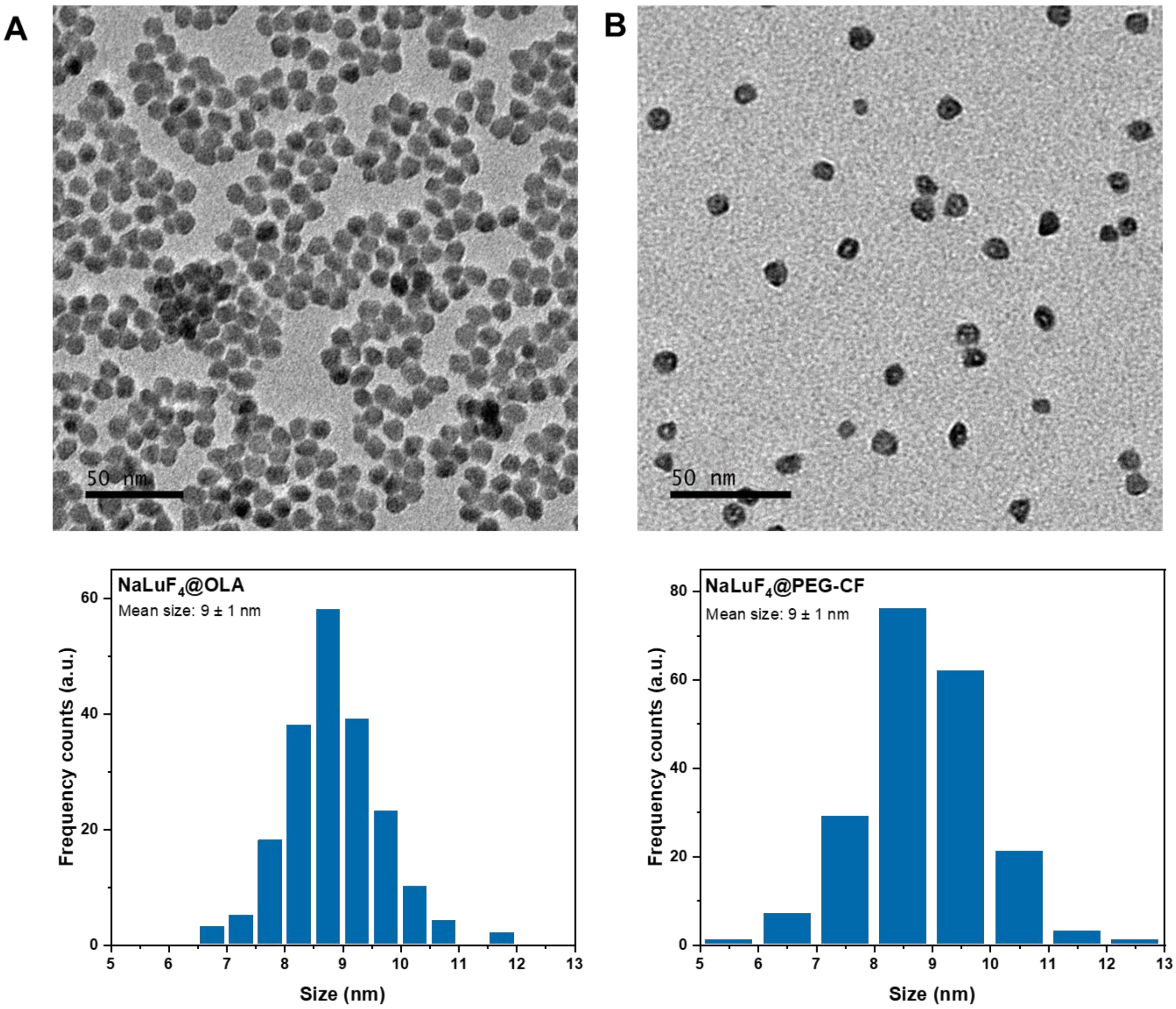

Figure S2. TEM images of $NaLuF_4$@OLA NPs (A) and $NaLuF_4$@PEG-CF NPs (B) and corresponding TEM size histograms.

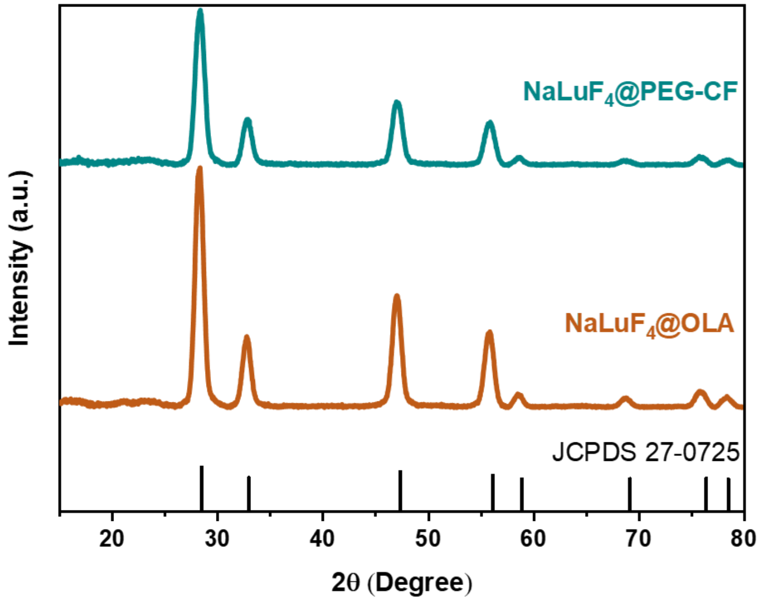

Figure S3. XRD of $NaLuF_4$ NPs before and after the ligand exchange with PEG-CF ligand, observing no difference in the pattern of cubic-phased $NaLuF_4$ (JCPDS 27-0725).

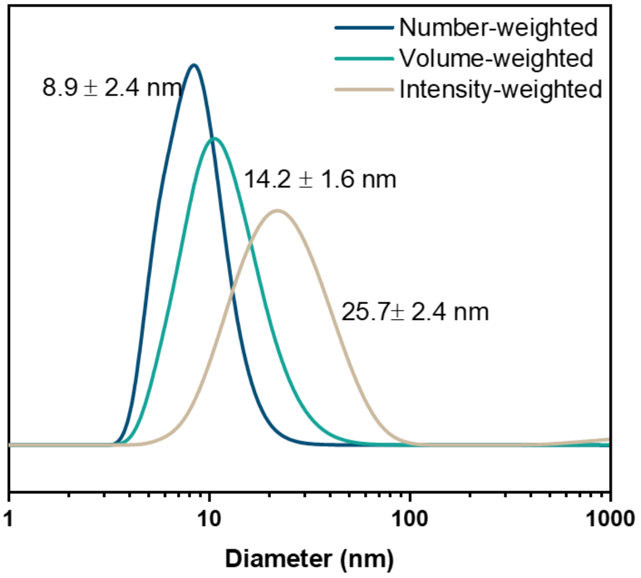

Figure S4. Hydrodynamic diameters (d_H) (mean value ± standard deviation, SD, from 3 measurements) as derived from DLS measurements for $NaGdF_4$@PEG-CF NPs in water.

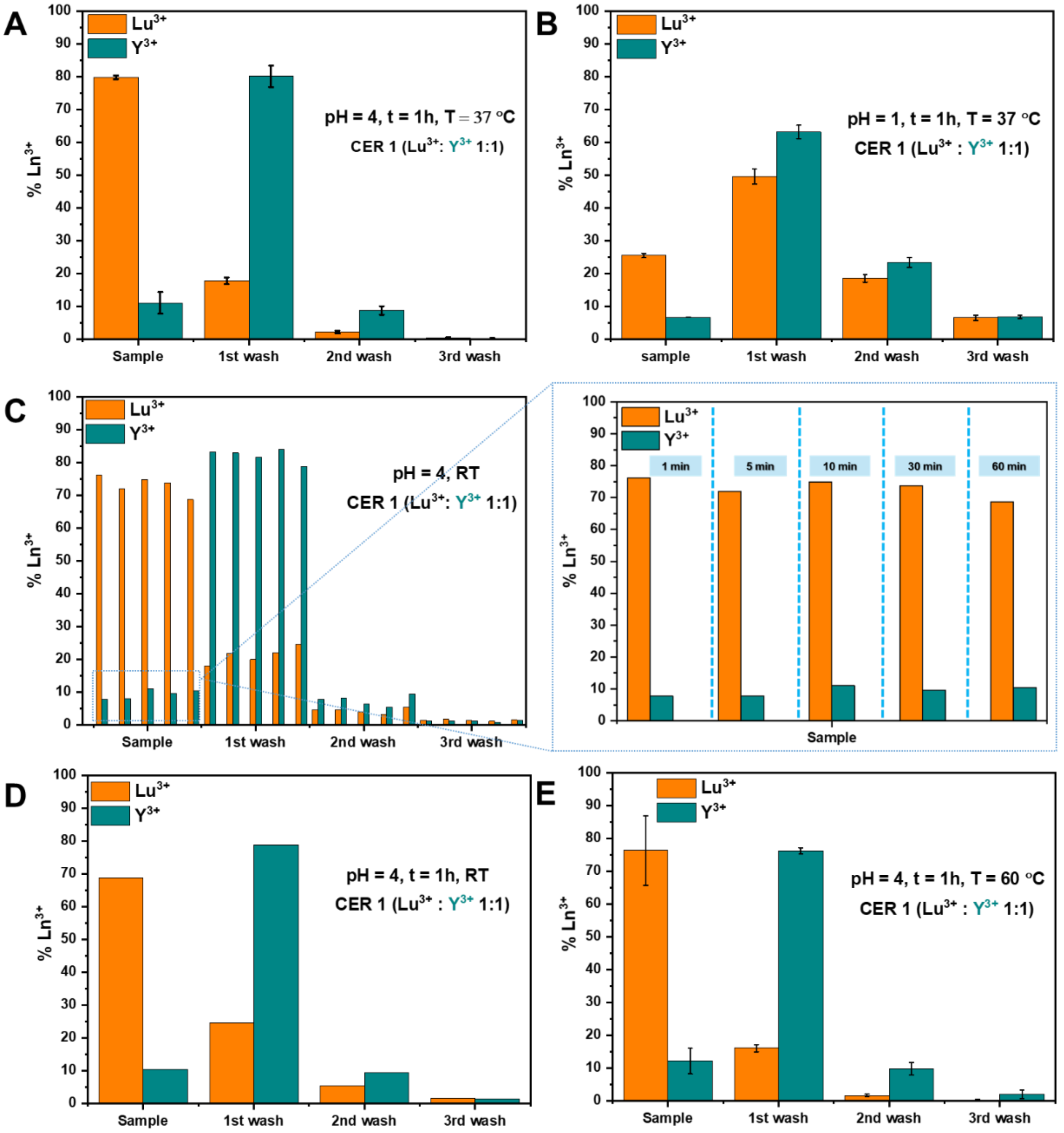

Figure S5. (A) CER of Y^{3+} on $NaLuF_4$, at Lu^{3+}:Y^{3+} ratio equal to 1 (CER 1), set at 37 °C temperature and pH=4 for 60 min. Elemental analysis of each washing step yields the concentration of incorporated Y^{3+} ions or the exchanged Lu^{3+} ions. Optimization of Y^{3+} insertion in $NaLuF_4$ by changing the (B) reaction pH to 1, (C) reaction times (1 min, 5 min, 10 min, 30 min, 60 min), (D) the reaction temperature of RT , and (E) 60 °C.

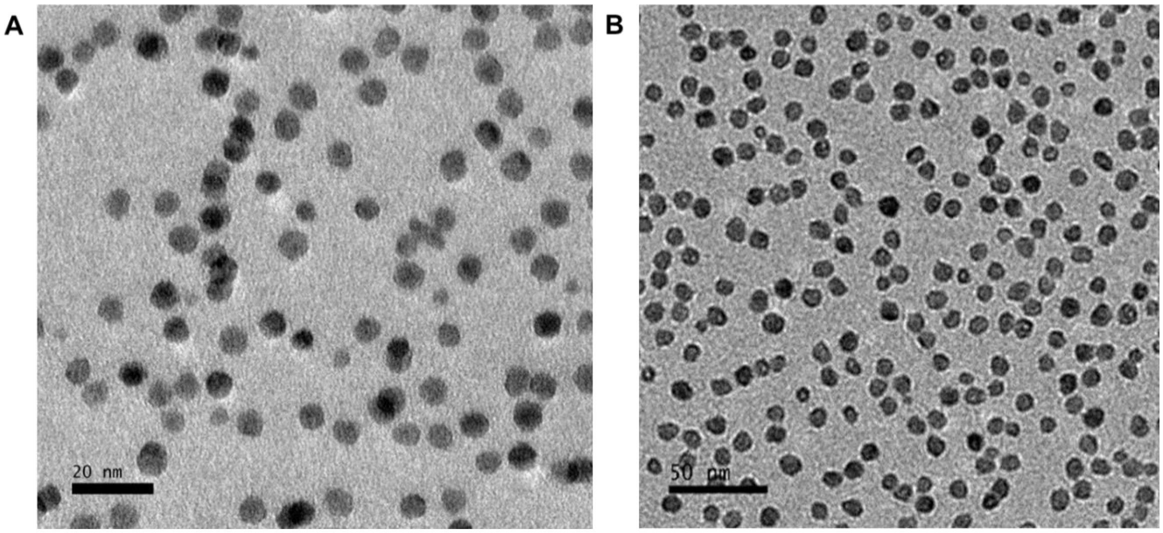

Figure S6. (A) TEM images of $NaLuF_4$ NPs and (B) the same NPs after CER with Y at Lu:Y ratio of 50:1 to give $NaLuF_4$:Y NPs

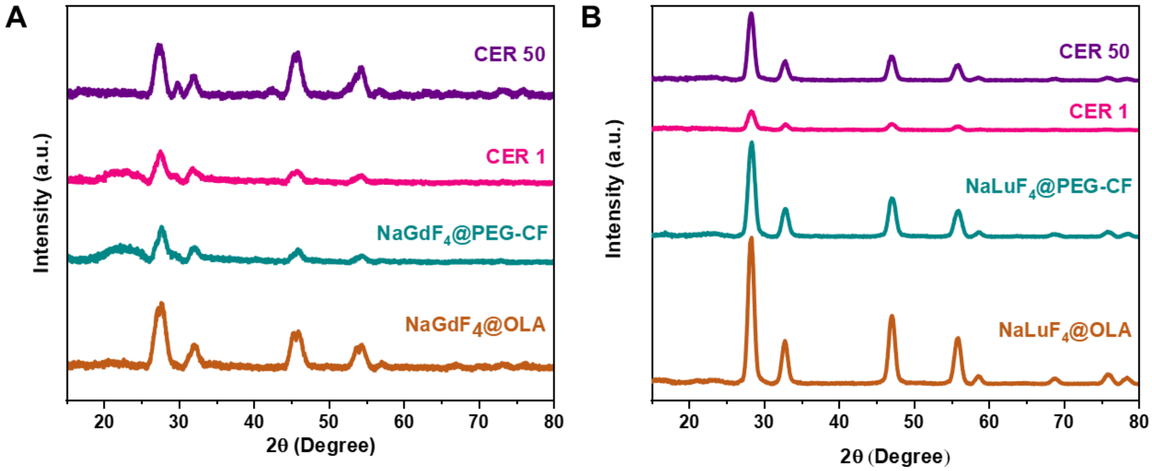

Figure S7. (A) XRD of $NaGdF_4$ NPs before and after CERs with Y at Gd:Y ratio of 50:1 (CER 50) and 1:1(CER 1), $NaGdF_4$@PEG-CF and $NaGdF_4$@OLA NPs; (B) XRD of $NaLuF_4$ NPs before and after CERs with Y at Lu:Y ratio of 50:1 (CER 50) and 1:1 (CER 1), $NaLuF_4$@PEG-CF and $NaLuF_4$@OLA NPs.

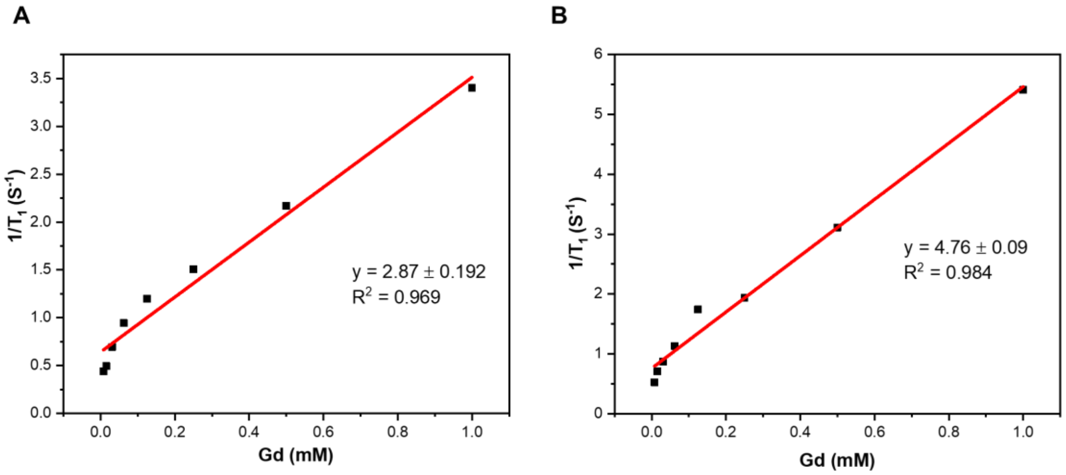

Figure S8. Linear calibration curves for T_1 relaxivity measurements of (A) $NaGdF_4$@PEG-CF NPs and (B) CER 50 reaction $NaGdF_4$@PEG-CF NPs and Y^{3+} for 2 h, whose inverse of relaxation times ($1/T_1$) were plotted against the Gd concentration respectively.

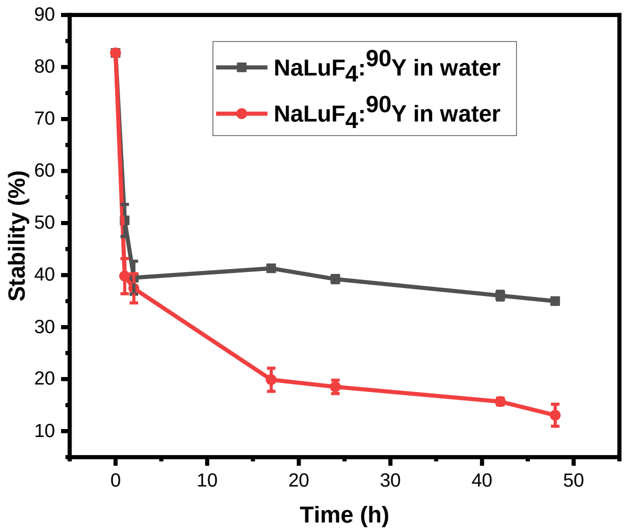

Figure S9. Stability of the $NaLuF_4$:^{90}Y in water and human serum, as determined by radio-TLC quantification (n=3).

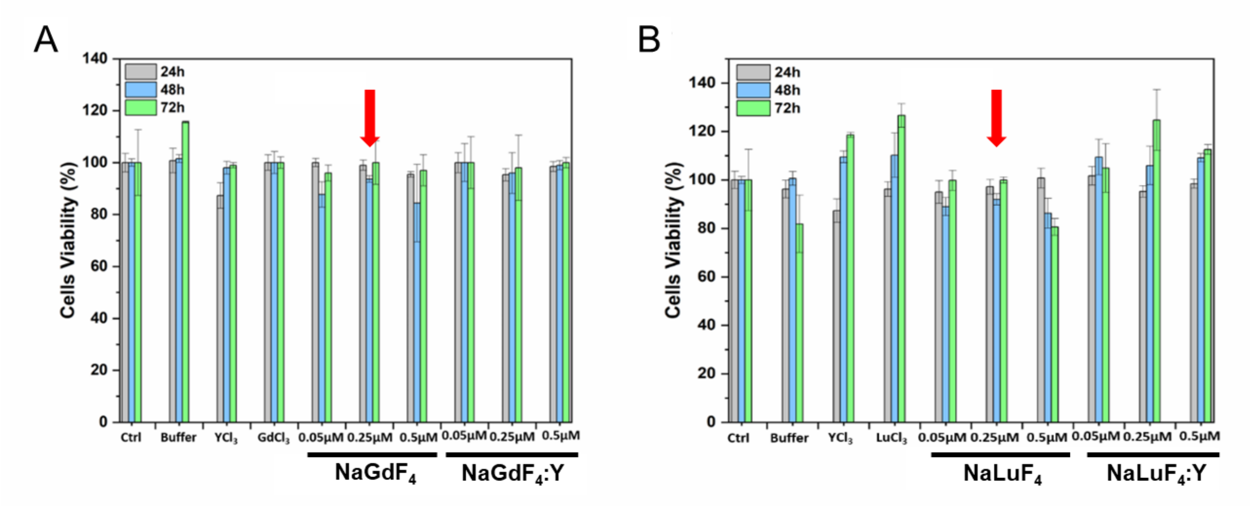

Figure S10. (A) Biocompatibility of $NaGdF_4$ and $NaGdF_4$:Y, along with all reagents used in the cation exchange process on A431 cells. The red arrow indicates the concentration of $NaGdF_4$ used for radiolabeling (n=3) (based on the concentration of Ln ions as obtained by ICP). (B) Biocompatibility of $NaLuF_4$ and $NaLuF_4$:Y, along with all reagents used in the cation exchange process on the A431 cells. The red arrow indicates the concentration of $NaLuF_4$ used for radiolabeling NPs (based on the concentration of Ln ions, Gd or Lu, as obtained by ICP) (n=3). The results obtained here are identical to the biocompatibility obtained for the U-87 cell line.

References

1. Milborne, B.; Arjuna, A.; Islam, M. T.; Arafat, A.; Layfield, R.; Thompson, A.; Ahmed, I., Yttrium-Enriched Phosphate Glass-Ceramic Microspheres for Bone Cancer Radiotherapy Treatment. *ACS Omega* **2024,** *9* (52), 50933-50944.
2. Banerjee, S.; Pillai, M. R.; Knapp, F. F., Lutetium-177 therapeutic radiopharmaceuticals: linking chemistry, radiochemistry, and practical applications. *Chem Rev* **2015,** *115* (8), 2934-74.
3. Yook, S.; Cai, Z.; Lu, Y.; Winnik, M. A.; Pignol, J. P.; Reilly, R. M., Radiation Nanomedicine for EGFR-Positive Breast Cancer: Panitumumab-Modified Gold Nanoparticles Complexed to the beta-Particle-Emitter, (177)Lu. *Mol Pharm* **2015,** *12* (11), 3963-72.
4. Guryev, E. L.; Volodina, N. O.; Shilyagina, N. Y.; Gudkov, S. V.; Balalaeva, I. V.; Volovetskiy, A. B.; Lyubeshkin, A. V.; Sen, A. V.; Ermilov, S. A.; Vodeneev, V. A.; Petrov,

R. V.; Zvyagin, A. V.; Alferov, Z. I.; Deyev, S. M., Radioactive ((90)Y) upconversion nanoparticles conjugated with recombinant targeted toxin for synergistic nanotheranostics of cancer. *Proc Natl Acad Sci U S A* **2018,** *115* (39), 9690-9695.

5. Viana, R. d. S.; Costa, L. A. d. M.; Harmon, A. C.; Gomes Filho, M. A.; Falcão, E. H. L.; Vicente, M. G. H.; Junior, S. A.; Mathis, J. M., 177Lu-Labeled Eu-Doped Mesoporous SiO2 Nanoparticles as a Theranostic Radiopharmaceutical for Colorectal Cancer. *ACS Applied Nano Materials* **2020,** *3* (9), 8691-8701.

6. Taejong Paik, A.-M. C., John L. Mikitsh, Joseph S. Friedberg, Daniel A. Pryma, and Christopher B. Murray, Shape-Controlled Synthesis of Isotopic Yttrium-90-Labeled Rare Earth Fluoride Nanocrystals for Multimodal Imaging. *Acs Nano* **2015,** *9*, 10.

7. Lemaître, T. A.; Burgoyne, A. R.; Ooms, M.; Parac-Vogt, T. N.; Cardinaels, T., Inorganic Radiolabeled Nanomaterials in Cancer Therapy: A Review. *ACS Applied Nano Materials* **2022,** *5* (7), 8680-8709.

8. Guo, Z.; Wang, X.; Han, Y.; Shen, S.; Tian, P.; Hu, Y.; Ding, Z.; Fu, Q.; Liu, Z., Targeted Radionuclide Therapy Activates Prodrugs for Treating Metastasis. *ACS Cent Sci* **2024,** *10* (12), 2321-2330.

9. Roy, I.; Krishnan, S.; Kabashin, A. V.; Zavestovskaya, I. N.; Prasad, P. N., Transforming Nuclear Medicine with Nanoradiopharmaceuticals. *ACS Nano* **2022,** *16* (4), 5036-5061.

10. Zhang, S.; Wang, X.; Gao, X.; Chen, X.; Li, L.; Li, G.; Liu, C.; Miao, Y.; Wang, R.; Hu, K., Radiopharmaceuticals and their applications in medicine. *Signal Transduction and Targeted Therapy* **2025,** *10* (1), 1.

11. Fallah, J.; Agrawal, S.; Gittleman, H.; Fiero, M. H.; Subramaniam, S.; John, C.; Chen, W.; Ricks, T. K.; Niu, G.; Fotenos, A.; Wang, M.; Chiang, K.; Pierce, W. F.; Suzman, D. L.; Tang, S.; Pazdur, R.; Amiri-Kordestani, L.; Ibrahim, A.; Kluetz, P. G., FDA Approval Summary: Lutetium Lu 177 Vipivotide Tetraxetan for Patients with Metastatic Castration-Resistant Prostate Cancer. *Clinical cancer research : an official journal of the American Association for Cancer Research* **2023,** *29* (9), 1651-1657.
